# Prevalence of high bloodpressure, hyperglycemia, dyslipidemia, metabolic syndrome and their determinants in Ethiopia: Evidences from the National NCDs STEPS Survey, 2015

**DOI:** 10.1371/journal.pone.0194819

**Published:** 2018-05-09

**Authors:** Yeweyenhareg Feleke Gebreyes, Dejuma Yadeta Goshu, Tedla Kebede Geletew, Terefe Gelibo Argefa, Theodros Getachew Zemedu, Kassahun Amenu Lemu, Feyissa Challa Waka, Alemayehu Bekele Mengesha, Fasil Shiferaw Degefu, Atkure Defar Deghebo, Habtamu Teklie Wubie, Mussie Gebremichael Negeri, Tefera Tadele Tesema, Yabetse Girma Tessema, Mulugeta Guta Regassa, Geremew Gonfa Eba, Misrak Getnet Beyene, Kissi Mudie Yesu, Girum Taye Zeleke, Yewondwossen Tadesse Mengistu, Abebe Bekele Belayneh

**Affiliations:** 1 College of Health Sciences, Addis Ababa University, Addis Ababa, Ethiopia; 2 Ethiopian Medical Association, Addis Ababa, Ethiopia; 3 Ethiopian Public Health Institute, Addis Ababa, Ethiopia; 4 Ethiopian Public Health Association, Addis Ababa, Ethiopia; 5 World Health Organization, Addis Ababa, Ethiopia; 6 Federal Ministry of Health, Addis Ababa, Ethiopia; Temple University School of Medicine, UNITED STATES

## Abstract

The prevalence of diabetes, dyslipidemias, and high blood pressure is increasing worldwide especially in low and middle income countries. World Health Organization has emphasized the importance of the assessment of the magnitude of the specific disease in each country. We determined the prevalence and determinant factors of high blood pressure, hyperglycemia, dyslipidemias and metabolic syndrome in Ethiopia. A community based survey was conducted from -April to June 2015 using WHO NCD STEPS instrument version 3.1. 2008. Multistage stratified systemic random sampling was used to select representative samples from 9 regions of the country. A total of 10,260 people aged 15–69 years participated in the study. Blood pressure (BP) was measured for 9788 individuals. A total of 9141 people underwent metabolic screening. The prevalence of raised blood pressure (SBP ≥140 and/or DBP ≥ 90 mmHg) was 15.8% (16.3% in females and 15.5% in males). The prevalence of diabetes mellitus (FBS ≥ 126 mg /dl) including those on medication was 3.2% (3.5% males and 3.0% females). The prevalence of impaired fasting glucose was 9.1% with ADA criteria and 3.8% with WHO criteria. Hypercholesterolemia was found in 5.2%, hypertriglyceridemia in 21.0%, high LDL cholesterol occurred in 14.1% and low HDL cholesterol occurred in 68.7%. The prevalence of metabolic syndrome using IDF definition was 4.8% (8.6% in females and vs. 1.8% in males). Advanced age, urban residence, lack of physical exercise, raised waist circumference, raised waist hip ratio, overweight or obesity, and total blood cholesterol were significantly associated with raised blood pressure (BP) and diabetes mellitus. Increased waist- hip ratio was an independent predictor of raised blood pressure, hyperglycemia and raised total cholesterol. Our study showed significantly high prevalence of raised blood pressure, hyperglycemia and dyslipidemia in Ethiopia. Community based interventions are recommended to control these risk factors.

## Introduction

According to the World Health Organization (WHO), 2017 report, Non- Communicable Diseases (NCDs) kill 40 million people. Three quarters of NCD deaths (28 million) occur in low and middleincome countries. Cardiovascular diseases account for most NCD deaths (17.7 million people) annually, followed by cancers (8.8 million), respiratory diseases (3.9 million), and diabetes (1.6 million) [1]. Globally blood pressure is the leading metabolic risk factor in terms of attributable deaths accounting for 18%, followed by overweight and obesity and raised blood glucose[2]. Chronic NCDs are rising fastest among lower–income countries [3–6]. The federal Ministry Health of Ethiopia established a National Strategic Action Plan for Non–Communicable Disease in Ethiopia (2014–2016), and developed national treatment guidelines and training materials on major NCDs like hypertension and diabetes. The national WHO STEPS survey was undertaken by the Federal Ministry of Health (FMOH) as part of a situational analysis of NCD risk factors to provide baseline data for subsequent interventions [7–10].

According to the Report of International Diabetes Federation (IDF) 2015 [11], the world- wide prevalence of diabetes mellitus among age group 20–79 was 8.8% (415 million people). WHO estimates that globally, high blood glucose is the third highest risk factor for premature mortality, after high blood pressure and tobacco use [12]. The regional prevalence of diabetes in the African population was estimated to be 2.1–6.7%. Ethiopia is estimated to have the fourth highest number of diabetes in the African region (1.3 million diabetes) [11].

The global prevalence of raised BP in adults aged 18 years and over was around 22% in 2014 (with highest prevalence being in Africa, at 30% for all adults combined [12](1. Hypertension has shown a rapid increase in prevalence affecting significant numbers of individuals in sub- Saharan Africa [13] (with a prevalence in the range of 25.4% to 41.1% in men and 27.2% to 38.7% in women [14] (. Different studies in Ethiopia showed a prevalence of hypertension of 19.6% (23.5% in urban population and 14.7% in rural/urban population), prevalence of in males and females was 20.6% and 19.2% respectively [15]. A study done in Tikur Anbessa specialized Hospital on stroke patients identified hypertension as a major risk factor in 69.3% of patients[16] (, Other studies also showed that hypertensive heart disease is the second most common diagnosis in the cardiac clinics[17–19]. A study from Nigeria, like Ethiopia, showed hypertension was more prevalent in the urban than rural dwellers with rates of 32.7% and 12.9% respectively[20].

Dyslipidemia (total cholesterol > 200 mg/dl, triglycerides > 150 mg /dl, LDL–C > 130 mg/l, HDL-C < 40 mg/dl; male, HDL–C < 50 mg/d; female) is one of the most important modifiable risk factors for the development of coronary heart disease[21]. According to a WHO estimate, globally the prevalence of raised total cholesterol is estimated to be 39%, and a third of ischemic heart disease is attributable to this risk factor[22]. The prevalence of dyslipidemias varies from country to country. In the USA,52% of adults had lipid abnormalities[23]. In Chinese subjects the prevalence of at least one type of abnormal lipid concentration was 64.4%[24]. In Nigeria, the prevalence of dyslipidemias ranged from 60% among apparently healthy Nigerians to 89% among diabetic Nigerians[25]. The prevalence of metabolic syndrome is estimated to be around 20–25 percent of the population and it doubled the risk to death and tripled the risk of cardiovascular disease compared to people without the syndrome[26,27]. Different studies have identified various sociodemographic factors like age, gender, place of residence and behavioral factors like excess alcohol intake, smoking and lack of physical activities as determinants of hypertension, diabetes and dyslipidemias[20,28,29].

So far there was no nationally representative study on NCD metabolic risk factors therefore this study as part of the National STEPS survey was aimed to determine the prevalence and determinant factors of high blood pressure, raised blood glucose and dyslipidemia and metabolic syndrome in Ethiopia.

## Methods and materials

### Study setting and period

The National WHO Steps survey was conducted in the 9 regions and two city administrations (Addis Ababa and Dire Dawa) of Ethiopia from mid-April to June 2015.

### Study population

The national target population for this survey included all men and women age 15–69 years old who had lived at their current place of residence for at least six months. This target population included all people who considered Ethiopia to be their primary place of residence regardless of their citizenship status. Individuals who were not a permanent resident of Ethiopia, who were institutionalized-including people residing in hospitals, prisons, nursing homes, and other similar institutions or residents whose primary residences are military camps or dormitories, critically ill, mentally disabled and those with some type of physical disability that is not suitable for physical measurement were excluded from this study.

### Sample size determination and sampling procedure

“A single population-proportion formula was used to determine the sample size. To adjust for the design effect, a complex sampling design effect coefficient of 1.5 was used to compute the sample size. To have an adequate level of precision for each age-sex estimate and place of residence, the sample was multiplied by the number of age-sex and place of residence groups for which the estimates were reported. Thus, Z-score = 1.96; proportion = 35.2%; this proportion is a comparative analysis on an estimated prevalence of raised blood pressure in Ethiopia according to countries by the WHO 2008, marginal error = 0.04 [30] design effect = 1.5; age-sex estimate and place of residence—sex estimate = 10 groups, and non-response rate = 20%. A total of 513 Enumeration Areas (EAs) were covered nationwide. Stratifying the sampling design, there were 404 rural EAs and 109 urban EAs. Taking into account the cost of the study and the level of precision—20 Households (HHs) per EA were selected using systematic random sampling and one eligible individual from each HH was selected using the Kish method (a random selection of eligible individuals at HH level). The final sample size was calculated to be 10,260 HHs (10,260 study participants)”. Reference 9 Annexed,WHO steps summary report,Ethiopia,2015 (see S1 Appendix). The survey was conducted using the WHO NCD STEPS instrument version 3.1. 2008[31] Step 1, Questionnaires based on interview on socio demographic data and behavioral status. Step 2, Physical measurement Step 3: Biochemical measurement of, fasting blood glucose, triglycerides, cholesterol, LDL and HDL (Laboratory procedures are annexed (see S2 Appendix). Additional optional questions were added to the instrument because they were locally relevant (see S3 Appendix)

### Data collection and management

Data collection was done simultaneously at the 9 regions and 2 city administrations by trained nurses and lab professionals during a face to face interview, using s standardized questionnaire. Data were collected digitally using personal digital assistants (PDAs, eSTEPs software was used to design and program the data collection tools in the PDAs). The use of the software and PDAs to collect the data helped to generate the final dataset quickly following the completion of data collection. The collected datasets were stored in the device as well as the memory card in redline markup language (rml) format. The rml files from the PDAs were transferred to the supervisors’ tablet computers via the Windows Mobile Device Centre. The files were then transferred to a central server located at Ethiopian Public Health Institute (EPHI), Addis Ababa via Internet file streaming system (IFSS) software. IFSS is an application that connects to and exchanges data with the server component. Supervisors managed tablets supported by internet (EVIDEO) and run the IFSS icon (IfssClientPC.exe) located in their desktop to send all the updated data files to central server by entering their user name and password. Finally, IFSS automatically performed packaging, file delivery and receipt of incoming files. Data was managed using Excel, SPSS and Stata software.

### Physical measurement and biochemical analysis

#### Physical measurements

Blood pressure and heart rate were measured for all survey participants whereas body weight, height, waist circumference, and hip circumference were measured for all survey participants except pregnant women. Body weight and height was measured with the electronic Growth Management Scale. It measures body weight and height, and calculates body mass index (BMI) (Body weight (kg)/ Body height/ (m^2^)). A BMI ≥ 25 indicates that a person is overweight, while a BMI ≥ 30 indicates that a person is obese. Waist circumference was measured by placing a tape measure around the bare abdomen at the midpoint between the lower margin of the last palpable rib and the top of iliac crest of the hip bone. Abdominal obesity was defined as a waist circumference of ≥94 cm for men and ≥80 cm women[32].

Blood pressure and heart rate measurements were taken three times on the right arm of the survey participants in a sitting position, using a Boso-Medicus Uno instrument with a universal cuff and automatic blood pressure and heart rate monitor. The mean of three measurements was taken for analysis. The measurements were taken after the participant had rested for 15 minutes, with three minutes of rest between the measurements (maximum deviation of cuff pressure measurement ± 3 mmHg, and of pulse rate display ± 5%).

Raised blood pressure was defined as: systolic blood pressure (SBP) ≥ 140 mmHg and/or diastolic blood pressure (DBP) ≥ 90 mmHg, or currently taking medication for hypertension. The percentage of respondents with treated and/or controlled raised blood pressure among those with raised blood pressure (SBP ≥140 and or DBP ≥ 90 mmHg) or currently taking medication for raised blood pressure was categorized as follows: For those taking medication as SBP <140 mmHg and DBP <90 mmHg (controlled hypertension) and SBP ≥140 mmHg and/or DBP ≥90 mmHg (uncontrolled hypertension) and for those not taking medication as SBP ≥140 mmHg and/or DBP ≥90 mmHg.

#### Biochemical analysis

Laboratory tests were performed for blood glucose, total cholesterol and HDL cholesterol using CardioCheck PA Analyser and for triglycerides using Cobas Integra 400 Plus (Roche Diagnostics GmbH, Mannheim, Germany) clinical chemistry analyzer. (Annexed) Concentrations of glucose (raised blood glucose level and percentage of respondents currently on medication for raised blood glucose), and lipid profile (total cholesterol, triglycerides, LDL and HDL cholesterol) were measured the next day after STEPS 1 and 2 of the data collection. Blood tests were performed for all survey respondents using a CardioChek PA Analyzer, after eight hours fasting.

WHO/IDF 2006[33] recommend that venous glucose should be the standard method for measuring and reporting glucose concentration in blood. However in recognition of wide use of capillary sampling, especially in under–resourced countries, conversion values for capillary plasma glucose are provided for post load values, fasting values for venous and capillary plasma glucose are identical[33].

Hyperglycemia is defined as plasma venous value: ≥ 7.0 mmol/L (126 mg/dl) and diabetes on medication, impaired fasting glucose (IFG—110–125 mg/dl (WHO criteria,) or 100–125 mg/dl, (ADA criteria), and normal blood glucose (< 110 mg /dl (WHO criteria) or < 100 mg/dl (ADA Criteria). dyslipidemia is defined as: (total cholesterol (≥200 mg/dl, triglycerides (≥ 150 mg/dl,LDL (≥130mg/dl and HDL cholesterol (40 mg/dl in male, < 50 mg/dl in female). In accordance with the IDF criteria, subjects were classified as having metabolic syndrome(MetS) if participants had abdominal obesity (defined as waist circumference of ≥94 cm for men and ≥80 cm women) plus two of any of the following risk factors: (1) raised TG level (≥150 mg/dL); (2) reduced HDLC (<40 mg/dL in males and <50 mg/dL in females); (3) raised blood pressure (systolic BP ≥130 or diastolic BP ≥85 mmHg) or treatment of previously diagnosed hypertension; (4) raised FG (≥100 mg/dL[32]

### Data analyses

Descriptive weighted analysis was done along with complex sample analysis, and bivariate and multivariate analysis were conducted for raised blood pressure, raised blood glucose, and raised total cholesterol and metabolic syndrome. Further statistical analyses were done by using chi-squared tests and logistic regression models. Chi-squared tests were used for testing association of independent variables with the dependent variable. All factors with a p-value <0.05 in the bivariate analysis were further entered into the multivariate logistic regression model to control for confounding effects. Odds ratios (OR) with 95% confidence intervals (CI) were calculated. Statistical significance was accepted at the 5% level (p<0.05).

### Ethical considerations

The study protocol was reviewed and approved by Institution Review Board of Ethiopian Public Health Institute (EPHI) and National Ethics Review Committee of Ministry of Science and Technology. Informed consent was obtained from each participant and consent obtained from parents and guardians for those participants between age 15–17. Adequate explanation was given to them on the survey procedure, risk, benefit, confidentiality, and the right to withdraw from the study. Individuals with high blood pressure, hyperglycemia and hypercholesterolemia were advised on prevention strategies by the survey team and also to visit the nearby health institution for further management.

## Results

### Prevalence of raised blood pressure and determinant factors

Overall BP was measured for 9788 individuals of which 8667(88.5%) were from rural areas and 5817(59.4%) were women. The overall prevalence of raised blood pressure (SBP ≥140 and/or DBP ≥ 90 mmHg) was 15.8% with slightly higher prevalence in females 16.3% versus (15.5%) in males. There was a progressive increase in prevalence of raised BP with increasing age of the participants. The urban prevalence of raised BP was 19.7% and rural prevalence was 14.9%. Among those with raised BP 42.7% had combined systolic and diastolic raised BP, 33.5% had isolated diastolic raised BP and 23.8% had isolated systolic raised BP. The prevalence of stage2 hypertension (> 160/90mmHg) was 4.4%. Of those with previous diagnosis of hypertension and on drug treatment, 53.4% had controlled BP (<140/90).

### Prevalence of hyperglycemia

As shown in Table 1 a total of 9141 people underwent metabolic screening, 3842 were males and 5299 were females. The overall prevalence of diabetes mellitus (≥ 126 mg /dl) and previously diagnosed diabetes on medication was 3.2% (3.5% males and 3.0% females), with the majority between the age of 35 to 65 years of age. Few participants were aged 65–69 years. Prevalence of hyperglycemia increased with increasing age group of 15–24 to 64 yrs. (2.8% to 4.9%). About 0.5% of the study participants were reported as having diabetes on medication. Among the risk factors 6.7% were overweight and 8% were obese, raised waist circumference occurred in 5.6% of all participants.

**Table 1:** Prevalence of raised blood pressure, hyperglycemia and their determinants, WHO STEPs study, 2015, Ethiopia.

Variables		Prevalence of raised blood pressure or taking medication		Prevalence of hyperglycaemia or currently taking medication	
		N	%	n	%
Sex	Male	673	15.5%	135	3.3%
	Female	1094	16.3%	155	3.0%
	Total	1767	15.8%	290	3.2%
Age Group	15–24	210	9.1%	38	2.6%
	25–34	425	15.0%	65	2.6%
	35–44	431	19.2%	62	3.9%
	45–54	319	22.2%	55	4.3%
	55–64	260	33.6%	51	4.9%
	65+	122	36.9%	19	4.6%
	Total	1767	15.8	290	3.2
Locality	Urban	659	19.7%	112	3.2%
	Rural	1108	14.9%	178	3.2%
Current alcohol use	Yes	662	16.4%	96	3.9%
	No	87	19.1%	14	4.5%
BMI	Underweight	258	9.5%	68	3.9%
	Normal	1125	17.0%	157	2.6%
	Overweight	257	36.3%	48	6.7%
	Obese	102	45.3%	16	8.2%
Quartiles of income	Q1	351	18.1%	45	2.9%
	Q2	286	14.6%	44	2.2%
	Q3	268	15.0%	41	2.6%
	Q4	333	17.6%	55	3.3%
Physical Activity Level	Low level	393	18.2%	76	4.8%
	Moderate Level	332	16.0%	68	4.2%
	High Level	1033	15.4%	144	2.6%
Raised waist circumference	Normal	1442	15.4%	180	2.9%
	Raised	303	43.3%	109	5.6%
Waist hip ratio level	Normal WHR	987	13.9%	142	4.3%
	Raised WHR	758	22.7%	289	3.2%
Raised Blood Pressure	Normal	NA	NA	202	3.1%
	Raised	NA	NA	88	3.7%
Raised blood glucose	No	1678	15.8%	NA	NA
	Yes	89	18.9%	NA	NA
Total Cholesterol level	<200	1494	16.0%	200	1.9%
	> = 200	176	20.6%	88	30.2%
	Total	1670	15.8%	288	3.3%

#### Table 2, showed subgroup analysis of blood sugar values of WHO Steps 2015, Ethiopia compared to IDF /ADA criteria

According to IDF criteria, about 939 (9.1%) of the study participants were found to have impaired fasting glucose (IFG 100–125 mg/dl) compared to WHO criteria (3, 8%), i.e intermediate hyperglycemia. Among these 8.8% of them were males and 9.6% were females; 10.4% lived in urban area and 8.9% lived in rural Ethiopia. Prevalence of IFG increased from 9.1% in age range of 15–24, to 12.1% in age range of 65 and above.

**Table 2:** Fasting blood glucose level (WHO/IDF, 2006 vs ADA 2003) by background characteristics, Ethiopia Steps 2015.

Variables		Blood glucose level Based on ADA/ IDF/WHO						Blood glucose level based on WHO			
		Normal (<100)		Impaired Fasting Glucose(100–125 mg/dl)		Hyperglycemia (> = 126 mg/dl)		Normal (<110)		Impaired Fasting Glucose(110–125 mg/dl)	
		n	%	n	%	n	%	n	%	n	%
Sex	Male	3075	87.7%	373	8.8%	127	3.5%	3298	92.7%	150	3.8%
	Female	4264	87.5%	546	9.6%	139	3.0%	4574	93.1%	236	3.9%
	Total	7339	87.6%	919	9.1%	266	3.3%	7872	92.9%	386	3.8%
Age Group	15–24	1717	88.1%	191	9.1%	38	2.8%	1832	93.8%	76	3.4%
	25–34	2278	89.6%	231	7.8%	61	2.6%	2412	93.9%	97	3.5%
	35–44	1596	85.6%	214	10.2%	61	4.1%	1720	91.5%	90	4.3%
	45–54	972	86.6%	144	9.2%	48	4.2%	1051	91.5%	65	4.3%
	55–64	530	83.9%	97	11.6%	42	4.5%	588	90.6%	39	4.9%
	65+	246	83.6%	42	12.1%	16	4.3%	269	88.9%	19	6.8%
	Total	7339	87.6%	919	9.1%	266	3.3%	7872	92.9%	386	3.8%
Locality of the respondents	Urban	1905	86.6%	251	10.4%	94	3.0%	2042	92.7%	114	4.3%
	Rural	5434	87.8%	668	8.9%	172	3.3%	5830	93.0%	272	3.7%
	Total	7339	87.6%	919	9.1%	266	3.3%	7872	92.9%	386	3.8%

Dyslipidemia: Total Cholesterol ≥ 200 mg/dl, Triglyceride ≥ 150 mg /dl, LDL–C > 130 mg/l, HDL-C < 40 mg/dl–Male, HDL–C < 50 mg/d–Female.

### Prevalence of raised total cholesterol

**Fig 1:**
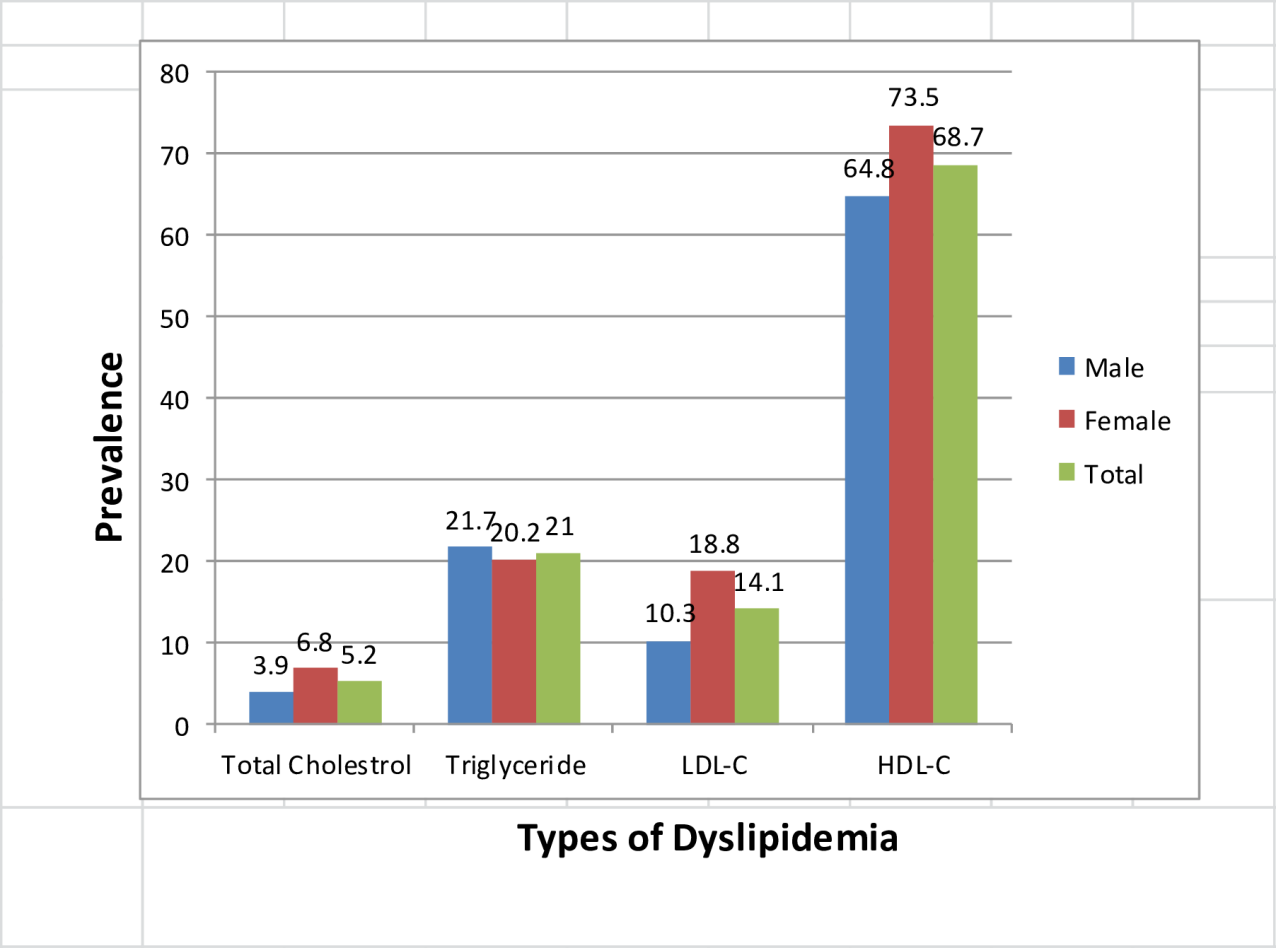
Prevalence of dyslipidemia, WHO STEPS survey, Ethiopia 2015.

**Fig 2:**
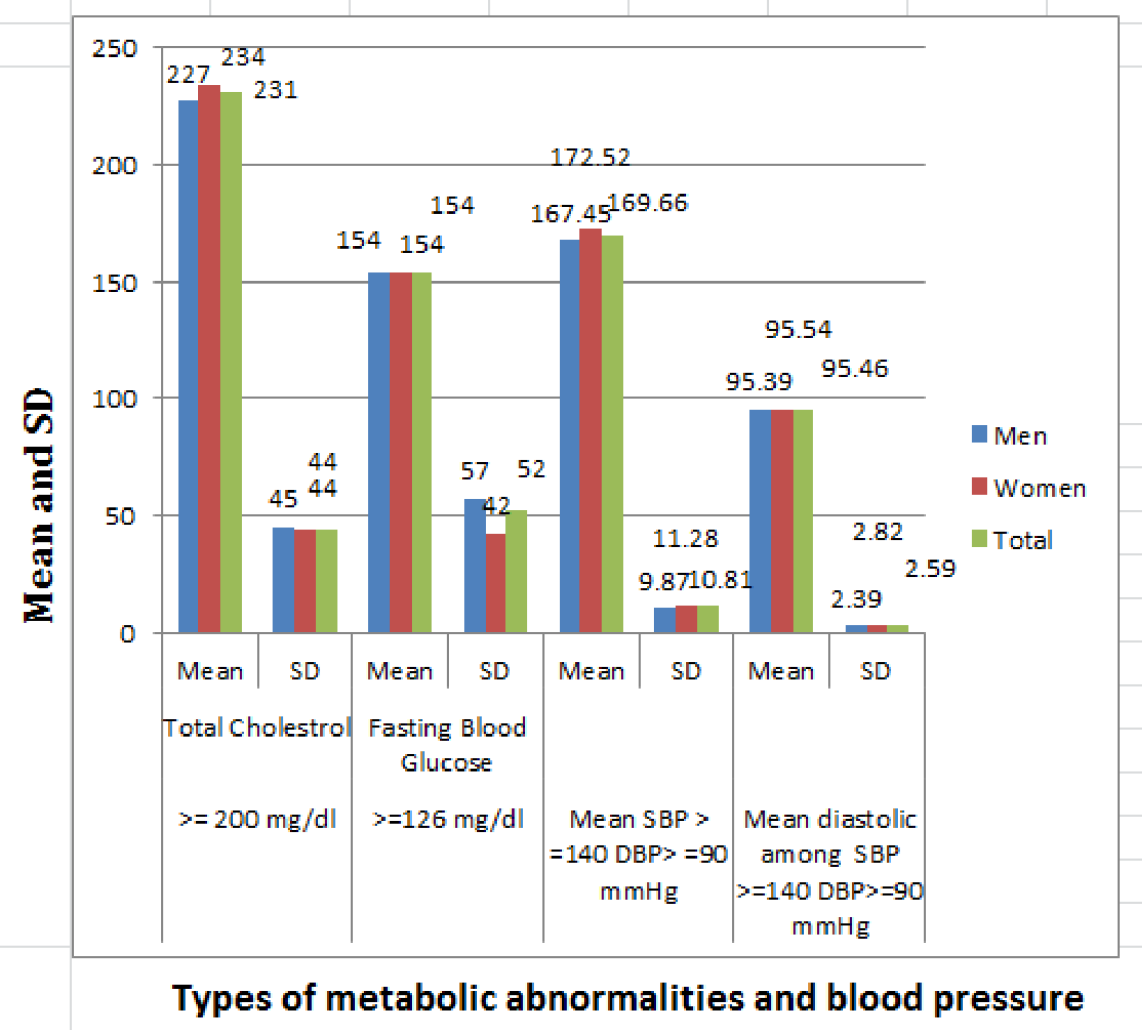
Mean and SD values of total cholesterol, blood glucose, diastolic and systolic blood pressure, Ethiopia NCD steps, 2015.

Hypercholesterolemia was found in 5.2%, of the study population which differs by sex, age group, area of residence, and other clinical and behavioral characteristics of study participants (Table 3, “Fig 1”). Low HDL–cholesterol and hypertriglyceridemia were the more prevalent dyslipidemias in the study participants. The mean levels of total cholesterol within participants having raised total cholesterol was 231±44mg/dl (“Fig 2”).

**Table 3:** Prevalence of raised total cholesterol level and metabolic syndrome, and their determinants WHO STEPS survey, Ethiopia 2015.

Variables		Prevalence of raised total Cholesterol (> = 200 mg/dl)		Prevalence of metabolic syndrome *	
		n	%	n	%
Sex	Male	165	3.9%	114	1.8%
	Female	421	6.8%	643	8.6%
	Total	586	5.2	757	4.8%
Age Group	15–24	84	4.1%	59	1.6%
	25–34	131	4.3%	144	3.6%
	35–44	141	6.2%	219	7.8%
	45–54	122	8.3%	171	9.8%
	55–64	81	8.9%	114	10.1%
	65+	27	3.3%	50	10.8%
	Total	586	5.2	757	4.8%
Locality	Urban	230	7.1%	437	11.7%
	Rural	356	4.8%	320	3.2%
Current alcohol use	Yes	236	7.1%	236	4.3%
	No	17	4.7%	47	8.3%
BMI	Underweight	99	4.8%	36	1.0%
	Normal	372	4.9%	374	3.9%
	Overweight	83	11.3%	240	29.7%
	Obese	29	8.4%	107	41.7%
Quartiles of income	Q1	76	4.2%	119	4.5%
	Q2	83	3.3%	123	4.6%
	Q3	112	6.1%	116	4.7%
	Q4	132	6.7%	188	6.8%
Physical Activity Level	Low level	140	6.8%	240	9.9%
	Moderate Level	121	6.7%	159	6.0%
	High Level	322	4.6%	356	3.6%
Raised waist circumference	Normal	371	4.6%	0	0.0%
	Raised	214	9.4%	757	39.8%
Waist hip ratio level	Normal WHR	339	5.0%	181	1.3%
	Raised WHR	246	5.8%	576	14.1%
Raised Blood Pressure	Normal	412	4.9%	278	2.1%
	Raised	174	7.1%	479	18.8%
Raised blood glucose (> = 126 mg/dl)	No	498	3.8%	663	4.3%
	Yes	88	46.5%	94	18.0%
Total Cholesterol level	<200	NA	NA	631	4.7%
	> = 200	NA	NA	122	12.3%
	Total	NA	NA	753	4.8%

*Metabolic syndrome (MetS) based on with the IDF criteria, subjects were classified as having MetS if participants had abdominal obesity (defined as waist circumference of ≥94 cm for men and ≥80 cm women) plus two of any of the following risk factors: (1) raised TG level (≥150 mg/dL); (2) reduced HDLC (<40 mg/dL in males and <50 mg/dL in females); (3) raised blood pressure (systolic BP ≥130 or diastolic BP ≥85 mmHg) or treatment of previously diagnosed hypertension; (4) raised FG (≥100 mg/dL

### Prevalence of metabolic syndrome

Table 3 depicts that the overall prevalence of metabolic syndrome in this study was 4.8% (757/ 8673); higher in females than in males (8.6% vs. 1.8%). Prevalence is higher in urban population than rural population (11.7% vs. 3.2%), increasing with increasing age group from 1.6% in age group 15–24 to 10.8% in age group 65 and above, prevalence was very high in obese patients compared to normal individuals (41% vs. 3.9%) and individuals with low level physical activities compared to high level physical activities (9.9% vs. 3.6%).

### Predictors of metabolic abnormalities

Demographic and clinical CVD risk factors were investigated as potential predictors for the metabolic abnormalities using unadjusted odds ratio (OR) and95% confidence interval (CI). All significant predictors were included in the multiple logistic regression analyses controlling for possible confounders to compute for adjusted OR and their 95% CI as an approximation of the adjusted relative risk. How well the model fit the data was estimated using the Hosmer–Lemeshow test of goodness of fit. All reported P-values were two tailed, and the level of statistical significance was set at 0.05. The results of both bivariate and multivariate logistic regression analyses of risk factors for the overall metabolic abnormalities are presented in Table 4. After controlling for confounders, our findings show that socio demographic and economic characteristics such as age group, gender and economic and other residential and clinical factors such as area of residence, waist circumference, and BMI were found to be independent predictors of metabolic abnormalities.

**Table 4:** Bivariate and multivariate analyses of demographic and clinical risk factors for raised blood pressure, raised blood sugar, raised total cholesterol level, Ethiopia NCD Steps 2015.

Variable characteristics		Raised blood pressure		Hyperglycemia		Total Cholesterol level > = 200	
		COR^1^ [95%CI]	AOR^2^ [95%CI]	COR^1^ [95%CI]	AOR^2^ [95%CI]	COR^1^ [95%CI]	AOR^2^ [95%CI]
		Bivariate	Multivariate	Bivariate	Multivariate	Bivariate	Multivariate
Sex	Male	1	1	1	1		
	Female	1.14(1.02,1.26)	1.06(0.92,1.22)	2.47(1.91,2.58)	2.25(1.63,3.11)	2.14(1.79,2.57)	1.97(1.96,1.98)
**Age Group**	15–24	1	1	1	1	1	1
	25–34	1.62(1.36,1.93)	1.47(1.19,1.82)	1.32(.88,1.97)	1.24(.76,2.03)	1.18(.89,1.56)	1.04(.75,1.42)
	35–44	2.54(2.13,3.03)	2.04(1.64,2.52)	1.75(1.16,2.63)	1.48(.90,2.45)	1.80(1.36,2.38)	1.49(1.08,2.04)
	45–54	3.26(2.69,3.94)	2.66(2.11,3.35)	2.51(1.65,3.82)	1.79(1.07,3.02)	2.56(1.92,3.41)	2.03(1.45,2.83)
	55–64	5.55(4.51,6.83)	4.01(3.11,5.18)	4.26(2.78,6.54)	3.20(1.88,5.45)	3.01(2.19,4.15)	1.98(1.35,2.901)
	65+	6.11(4.68,7.96)	4.88(3.55,6.73)	3.55(2.02,6.24)	3.04(1.58,5.85)	2.16(1.38,3.39)	1.70(1.02,2.84)
**Locality**	Rural	1	1	1	1	1	1
	Urban	1.76(1.58,1.96)	1.27(1.10,1.48)	1.68(1.32,2.14)	1.26(.92,1.75)	1.87(1.58,2.23)	1.23(.98,1.54)
**BMI**	Underweight	1	1	1	1	1	
	Normal	1.67(1.44–1.93)	1.60(1.34,1.89)	.81(.60,1.09)	.64(.46,.91)	1.36(1.09,1.71)	1.20(.93–1.55)
	Overweight	4.11(3.36–5.02)	2.39(1.83,3.10)	2.20(1.51–3.22)	1.12(.67,1.86)	2.78(2.05,3.77)	1.21(.82–1.79)
	Obese	6.79(5.04–9.15)	3.07(2.11,4.48)	2.51(1.43,4.41)	.93(.46,1.92)	3.37(2.16,5.25)	1.22(.71,2.09)
**Quintiles of income**	Q1	1	1	1	1	1	
	Q2	.82(.67,.99)	.80(.65,.99)	1.19(.80,1.77)	1.20(.78,1.85)	1.43(1.09,1.87)	1.48(1.11,1.97)
	Q3	1.047(.81,1.3)	1.01(.76,1.34)	.86(.45,1.65)	.71(.35,1.45)	2.17(1.57,3.01)	2.37(1.68–3.34)
	Q4	1.24(1.01,1.53)	1.14(.90,1.44)	1.20(.73–1.97)	1.08(.64,1.82)	1.79(1.31,2.44)	1.61(1.15,2.24)
	Q5	.98(.79,1.21)	.77(.61,.99)	1.62(1.07,2.44)	1.50(.96,2.34)	1.39(1.00,1.92)	1.09(.77,1.56)
Physical Activity Level	Low	1.37(1.23,1.59)	.96(.78,1.17)	1.91(1.44,2.53)	1.06(.71,1.57)	1.61(1.31,1.97)	.99(.74,1.32)
	Moderate	1.17(1.02,1.34)	.94(.80,1.118)	1.73(1.29,2.32)	.74(.52,1.05)	1.40(1.12,1.73)	.87(.68,1.11)
	High L	1	1	1	1	1	1
Frequency of adding salt	Never Add	1	1	1	1	1	1
	Sometimes	1.12(.98,1.28)	1.04(.89,1.22)	1.73(1.20,2.50)	1.68(1.11,2.54)	1.40(1.00,1.72)	1.19(.92,1.55)
	Usually	.99(.83,1.17)	.87(.71,1.07)	1.99(1.32,3.02)	1.80(1.12,2.88)	1.91(1.45–2.51)	1.84(1.37,2.47)
Raised waist circumference	Normal	1	1	1	1	1	1
	Raised	2.69(2.38,3.02)	1.56(1.30,1.88)	2.49(1.95,3.18)	1.16(.79,1.71)	2.51(2.10–2.99)	1.647(1.27,2.14)
Waist hip ratio level	Normal	1	1	1	1	1	1
	Raised	1.87(1.68,2.08)	1.25(1.09,1.44)	2.13(1.68,2.69)	1.55(1.14,2.12))	1.61(1.36,1.91)	1.02(.82,1.28)
Raised Blood pressure	Normal	NA	NA	1	1	1	1
	Raised	NA	NA	1.96(1.52,2.54)	1.26(.92,1.73)	1.89(1.57,2.27)	1.32(1.06,1.65)
Raised Blood glucose (WHO)	<126	1	1	NA	NA	1	1
	> = 126	2.03(1.57,2.61)	1.25(.91,1.72)	NA	NA	6.98(5.35,9.11)	4.2(3.02,5.84)
Raised total cholesterol	Normal	1	1	1	1	NA	NA
	Raised	1.68(1.40,2.01)	1.35(1.08,1.68)	6.98(5.35–9.11)	4.36(3.15,6.05)	NA	NA

^1^ Crude odds Ratio (COR).

^2^ Adjusted Odds ratio (AOR).

Among the non-modifiable risk factors, despite the preponderance of the female population in our samples, the prevalence of metabolic abnormality was not statistically different between the sexes; however, individuals aged ≥65 years were at increased risk of developing the metabolic abnormality (Table 4). On analysis of various socio-demographic and behavioral factors: age, urban residence, tobacco use, lack of physical exercise, alcohol intake, added salt, BMI were associated with raised BP. On multivariate logistic regression analyses urban residence, age, lack of physical exercise and BMI were strongly associated with raised BP (Table 4). Similarly, advanced age, urban residence, adding salt to food, low physical activities, raised waist circumference, raised waist hip ratio, overweight or obese, and having raised blood glucose were significantly associated with diabetes mellitus, and hypercholesterolemia. Our results also show that increased waist/hip ratio was independently predictive of the raised blood pressure, raised blood sugar and raised total cholesterol with (adjusted OR 1.25, 1.55 and 1.02) respectively. Similarly increased waist circumference was independently predictive of the raised blood pressure and raised total cholesterol with (adjusted OR 1.56, 1.64)) respectively.

## Discussions

Our study is the first national community based survey on NCDs and one of the few WHO STEPS Surveys in Africa, which applied all the three instruments of STEPS tools. Our main findings showed prevalence of high blood pressure in 15.8%, with higher prevalence in urban population versus rural population (19.7% vs 14.9%), Vantu NCD steps survey in 2012 showed higher prevalence compared to our report (28.6%)[34]. Diabetes occurred in 3.2%, which is slightly lower than the IDF Country estimate for Ethiopia 2015 (3.4%)[11] which was derived by extrapolation from neighboring countries. Impaired fasting glucose occurred in 9.1% (ADA criteria) or in 3.8% (WHO criteria). Hypertriglyceridemia and low HDL cholesterol were more prevalent dyslipidemias, and metabolic syndrome occurred in 4.8% of study participants. Our study showed a lower prevalence of metabolic syndrome compared to reports from India (35.8%- 45.3%) and China (30.5%-31.5%)[35,36]. The prevalence of all metabolic cardiovascular risk factors was higher in urban than rural populations and progressively increased with advancing age. Finding obtained from community based studies from Indian population also showed CVS risk factors significantly increased with advancing age[37]. Increased waist hip ratio was independently predictive of the raised blood pressure, raised blood sugar and raised total cholesterol with (adjusted OR 1.25, 1.55 and 1.02) respectively. Similarly increased waist circumference was independently predictive of the raised blood pressure and raised total cholesterol with (adjusted OR 1.56, 1.64)) respectively. Previous report from Northern Ethiopia also showed gender, age and area of residency and education as risk factors for CVD[38]

Our study revealed considerably high prevalence of raised BP (15.8%), and is much lower than that of the 2014 WHO national estimate for raised BP in adults >18 years (28.8%) and the pooled national prevalence estimate (19.6%), but higher than that of Butajira (12% in men and 8% in women)[12,15,39].This difference is possibly attributed to the difference in place of residence and age composition of the study population. In this survey the prevalence of raised BP has been shown to increase with age with the highest prevalence being in those above 65 years of age. Elevation in both systolic and diastolic BP is the commonest form followed by isolated diastolic BP elevation. Other studies also showed more isolated diastolic BP than isolated raised systolic BP in younger individuals below 40 years of age[40,41] and the higher proportion of younger population in the study might have resulted in higher proportion of diastolic raised BP in our study. The Framingham Heart Study showed that DBP was a better predictor of future coronary heart disease events than SBP in adults <50 years of age; the reverse was true after 50 years of age underlying the importance of isolated diastolic hypertension[42]. There is an observed difference between rural and urban respondents. This has been demonstrated in several other studies related to more sedentary life style in urban dwellers[28,43] The urban prevalence of raised BP in our study is considerably lower than that of urban Addis Ababa (31.2% males; 28.5% female) and Bahr Dar25.1%[42,44]. A recent Study also showed higher prevalence (25%) among 2716 adults in Addis Ababa[45]. These differences can be explained by the differences in the level of urbanization with corresponding difference in the determinants of raised BP.

The prevalence of raised BP in our study is higher in females but on multivariate analysis there was no statistically significant difference in the prevalence of raised BP between males and females. Prevalence of raised BP was found to be higher in males than females in several studies until menopause [46]. The gender disparity in raised BP is believed to be due to difference in biological and behavioral factors including hormonal difference, obesity, cigarette smoking, alcohol consumption and physical activities. The apparently high prevalence of raised BP in females in our study could be due to significant difference in level of physical activity; men (318.2 minutes, 95% CI: 302.7–333.7) and women (236.2 minutes, 95% CI:223.1–249.3) and higher prevalence of obesity in females (2.0 versus 0.5%). Treatment of hypertension was found to be lower than that reported from other low income countries (2.8% versus 31.7%)(36),[47]. The survey identified several determinants of high BP; the strongest being age. Other determinants included place of residence, alcohol intake, cigarette smoking, khat use, BMI, raised BMI and FBG. Similar determinants were also reported in other studies[20,29,39] In the current community survey, the prevalence of diabetes mellitus was 3.2% (3.3% in males and 3.0% in females).

This was an epidemiological survey and repeat testing was not done, The prevalence of diabetes and pre diabetes was lower compared to reports from urban slums of Bangalore (12.33%, 11.57%) respectively[48]. They studied urban population age 35 and above whereas we included both rural and urban population age 15 -69years. The inclusion of younger people in our survey may have reduced the prevalence. The prevalence was more among the females than males similar to the later report. A population based prevalence survey in Austria showed a higher overall diabetes prevalence of 7.4%. Prevalence in men was 8.0% and in women was 6.8%. In addition, 17.4% of men and 15.45 of women had IGT or IFG[49]. Malawi STEPs survey 2009 [50]. showed a prevalence raised blood pressure in 32.9%, diabetes in 5.6%, and raised cholesterol in 8.7% compared to ours (15.8%, 3.2%, 5.2%) respectively.

Impaired fasting glucose is a prediabetes state. In the current study the prevalence of impaired fasting glucose (IFG) was 9.1% (ADA criteria) vs. 3.8% (WHO Criteria). Use of the ADA criteria more than doubled the prevalence of IFG. There are a number of differences in several diagnostic criteria of glucose intolerance between WHO and ADA. Increasing fasting plasma glucose in a non-diabetic range is associated with fatal and non-fatal cardiovascular disease[51,52]. WHO recommended the fasting plasma glucose cut point for IFG should remain at 6.1 mmol/L(110 mg/dl) (WHO,2006, Recommendation 4 and 5) and ADA proposed a lower blood sugar values (100 mg/dl) [33]. Incidence rates of diabetes are important consideration. Mauritius and Pima Indian data indicated that risk of progression of IFG in the additional people identified by ADA criteria is half that of WHO criteria (15% versus 30%) over 5 years [53,54]. Our study also showed a significant difference in the prevalence of IFG (9.1% vs 5.4%) compared to WHO vs ADA criteria[33] and identified risk groups for diabetes and cardiovascular diseases. In our study the prediabetes state was more common in females, urban dwellers, prevalence increased with advancing age, higher in overweight and obese individuals, and individuals with low physical activities. Such findings are warrants an early intervention to halt the progression to diabetes and cardiovascular disease.

Dyslipidemias are the major cardiovascular risk factors. Our study showed that among dyslipidemias, hypertriglyceridemia and low HDL-C occurred in 21% and 68% of participants respectively. The most prevalent dyslipidemia was low HDL- cholesterol, which suggests that participants may be prone to cardiovascular disease. A study from São Paulo Brazil also showed high prevalence of low HDL = C (59.74%)[55]. The ICMR-INDIAB study[56] reported 13.9% had hypercholesterolemia, 29.5% had hypertriglyceridemia 72.3% had low HDL and 11.8% had high LDL. Seventy-nine (79%) had abnormalities in one of the lipid parameters. Similar trends were seen in our study.

As already mentioned above, we have applied IDF definition and found a prevalence of metabolic syndrome of 4.8% of study participants. The prevalence of metabolic syndrome is variable depending on the definitions of metabolic syndrome. The prevalence of metabolic syndrome in African populations ranges from as low as 0% to as high as about 50% or even higher in certain population setting[57], In study conducted in Malaysia the prevalence of metabolic syndrome according to IDF, NCEP ATP III and WHO definitions were 22.9%, 16.5% and 6. 4% respectively[58]. Several reports showed a high prevalence of metabolic syndrome compared to our report (China 35.8% to 45.3%, India 35.8% to 45.3%)[35]. In our study metabolic syndrome was frequently occurred with its determinants like raised waist circumference, high blood pressure, raised blood glucose, hypercholesterolemia, and a low level of physical activity.

In general overweight and obesity are associated with an increased risk of developing hypertension and diabetes[59–61]. In multivariate analysis, our results also showed that increased waist hip ratio was independently predictive of the raised blood pressure, hyperglycemia and raised total cholesterol (adjusted OR 1.25, 1.55 and 1.02) respectively. Similarly, increased waist circumference was independently predictive of the raised blood pressure and raised total cholesterol with (adjusted OR 1.56, 1.64)). Such a finding is alarming in resource-limited settings like Ethiopia where screening programs for CVD risk factors are not a priority due to the high burden of infectious diseases.

### Strength and limitations

Our study is the first nationwide large community based survey on metabolic risk factors of cardiovascular disease. In addition, the study findings can be generalized to the population because of the large sample size, sampling procedures, and high response rate of study participants. Some of the limitations were since it was a community survey, classification of diabetes and repeat blood glucose determination was not done as per recommendation of ADA.

Conclusions and Recommendations: The current community based WHO steps survey identified the prevalence of high blood pressure, diabetes, in 15.8% and 3.2% respectively. Hypertriglyceridemia and a low HDL were the most common dyslipidemias. Prevalence of IFG was 9.1% using ADA criteria more than double those identified by WHO criteria. Metabolic syndrome occurred in 4.8% of study population. Raised blood pressure, diabetes, dyslipIdemias, metabolic syndrome were more common in females, urban population and with advancing age. Increased waist hip ratio was an independent predictor of the raised blood pressure, hyperglycemia and raised total cholesterol; In general we recommend establishing a community based screening and intervention strategies on NCD’s modifiable risk factors and establishing efficient and sustainable health education system on life style modification.

## Supporting information

S1 Appendix NCD WHO STEPS summary report,Ethiopia, 2015. (PDF)

S2 Appendix 5.5–010 SOP for triglycerides. (DOC)

S3 Appendix NCD WHO STEPS English questionnaire. (PDF)
