# Supplementary material for: Prevalence of high bloodpressure, hyperglycemia, dyslipidemia, metabolic syndrome and their determinants in Ethiopia: Evidences from the National NCDs STEPS Survey, 2015
Source: PLoS One. 2018 May 9;13(5):e0194819. doi: 10.1371/journal.pone.0194819 (PMC5942803; doi:10.1371/journal.pone.0194819)
Supplement: S1 Appendix — (PDF) [file pone.0194819.s001.pdf]

STEPS SURVEY ON RISK FACTORS FOR NON-COMMUNICABLE DISEAES AND PREVALENCE OF SELECTED NCDs, ETHIOPIA

SUMMARY REPORT

EPHI

FMOH

Ethiopian Public Health Institute
Addis Ababa
December 2016

SUMMARY REPORT ON ETHIOPIA STEPS SURVEY ON RISK FACTORS FOR CHRONIC NON-COMMUNICABLE DISEASES AND PREVALENCE OF SELECTED NCDs

EPHI

FMOH

World Health Organization

Ethiopian Public Health Institute
Addis Ababa
December 2016

TABLE OF CONTENTS

INTRODUCTION 3
METHODS AND MATERIALS 4
ETHIOPIA STEPS SURVEY 2015 FINDINGS 4
CONCLUSIONS AND RECOMMENDATIONS 8
ACKNOWLEDGEMENTS 11
LIST OF CONTRIBUTORS 12

This report presents summary findings of the 2015 Ethiopia STEPS survey, which was implemented by the Ethiopian Public Health Institute in collaboration with the Ethiopian Ministry of Health. World Health Organization (WHO) provided technical assistance for the survey. The STEPS survey was used to measure chronic disease risk factors and the prevalence of selected NCDs in Ethiopia.

Additional information about the Ethiopia 2015 NCD STEPS survey can be obtained from the Ethiopian Public Health Institute (EPHI), Gulele Arbegnoch Street, Gulele Sub City, Addis Ababa, Ethiopia. Telephone: +251.11.275.4647; Fax: +251.11.275.4744; website: http://www.gov
STEPS country focal point name: Abebe Bekele Belayneh; email: abebe1277belay@gmail.com
Information about the WHO STEPS can be obtained from WHO STEPS Team [Steps@who.int]

Introduction

In countries across the world, regardless of geographic location, size of the population or stages of social and economic development, non-communicable diseases (NCDs) are responsible for high proportion of deaths and disabilities. As the leading cause of death globally, NCDs particularly cardiovascular diseases (CVDs), diabetes, chronic respiratory diseases and cancer were responsible for 38 million (68%) of the World's 56 million deaths in 2012. Almost three quarters of all NCD deaths (28 million), and the majority of premature deaths (82%) occur in low-and middle-income countries (LMIC). The cumulative economic losses in LMIC between 2011 and 2025 has been estimated US$7 trillion. This figure exceeds the annual US$ 11.2 billion cost of implementing a set of high-impact interventions to reduce NCD burden. In many low-income countries, non-communicable diseases were not priority public health agenda due to the heavy burden of communicable diseases, and other competing issues with no exception to Ethiopia. There is scarcity of epidemiologic data that describe major non-communicable disease and their risk factors in sub –Saharan African countries. Likewise, except very few studies in some pocket areas, there was no representative NCD risk factor survey undertaken in Ethiopia. Despite the limitations in the health management information system (HMIS) of Ethiopia, non-communicable diseases such as hypertension and diabetes mellitus appear on the list of leading causes of morbidity and mortality in the hospitals and regional health bureaus across the country. The EDHS 2011 results on behavioural risk factors (tobacco use, alcohol consumption, and khat chewing) showed that 7 % of men use tobacco products; 45% of women and 53% of men reported drinking alcohol in their lifetime; 11% of women and 28% of men reported that they have ever chewed khat. In one of the administrative zones of Ethiopia, a population-based cross-sectional survey (STEPS) was conducted in Gilgel Gibe, one of the Ethiopian demographic and surveillance sites in 2009 and a random sample of 4,469 individuals age 15-64 years were studied. Overall prevalence of NCDs was 8.9%. Prevalence of 3.1% for diabetes, 9.3% for hypertension, 3.0% for cardiovascular diseases, 1.5% for asthma and 2.7% for mental illness were described. As the trend of NCD burden is increasing in the health facilities of the country, the need to conduct a comprehensive survey has been given priority by FMOH. The basis of disease prevention is the identification of the major common risk factors and their prevention and control as the risk factors of today are the diseases of tomorrow. Additionally, information from this study provides baseline data to assess disease trends and impact of various interventions. This is the first national NCD risk factor survey against which future surveys can be based to assess impact and effectiveness of national prevention and control efforts for NCDs and their risk factors. The Ethiopia NCD STEPS survey provides baseline data of risk factors for non-communicable diseases (NCDs). The survey was conducted with the objectives of assessing behavioural and biological risk factors for major chronic non-communicable diseases (NCDs) and prevalence of selected NCDs to establish baseline information for policy and program development.

METHODS AND MATERIALS

A community based cross sectional study was conducted in accordance to the WHO a step-wise approach to the surveillance of NCD risk factors. The survey was conducted across the country between April and June 2015. The data collection processes included three steps Step 1:This step comprised a questionnaire to gather demographic and behavioural characteristics of the study population, Step 2: Physical measurement was done to build on the core data in step 1 and to determine proportion of the study population with raised blood pressure, overweight and obesity, and Step 3: Biochemical measurements were undertaken to build on the core data in step 1 and step 2 to measure proportion of the study population with diabetes, raised blood glucose and abnormal lipid level. In addition to core and expanded modules, some optional modules were included in each of the three steps. Data were collected digitally using personal digital assistants (PDAs) from which data were transferred to central server using internet file streaming system (IFSS) and exported to Microsoft Excel on personal computers. Data was cleaned using SPSS and Stata and analysed using Epi Info version 3.5.4. Descriptive weighted analysis was done along with complex sample analysis, and bivariate and multivariate analysis was conducted for diabetes and hypertension.

ETHIOPIA STEPS SURVEY 2015 FINDINGS

Sociodemographic profiles

Of the sampled 10,260 individuals, 9,800 respondents, age 15-69 years were involved in the survey and the response rate was 95.5%. Of the total 9,800 respondents of STEPs survey, about six in ten were women. Regarding education level by age, the younger group was more likely educated compared with respondents in the older age group. Of all the respondents 49.4 % had no formal education, while 28.8 % attended formal education with less than primary level. Majority of the respondents (67.3 %) were currently married; nearly 10% were employed; 71% of study participants reported that their annual income was less than 12,000 Birr.

Behavioural Risk Factors: STEP 1

The data collected on behavioural characteristics showed that about 4.2% of the survey participants were current smokers (men 7.3%, women 0.4%). Among all current smokers of both sexes, 82.8% of them smoked tobacco daily. Ten percent were exposed to second-hand smoke at home whereas 13% in the workplace. With regard to alcohol consumption, nearly 41% had consumed alcohol during the past 30 days prior to the survey. The proportion of men who consumed alcohol (46.6%), was higher than that of women (33.5%).The average number of days per week on which fruit and vegetable consumed was 0.9 and 1.5, respectively. More than ninety-eight percent of the population consumed fewer than five servings of fruit and vegetables daily. About six percent of the study population did not meet WHO recommendations on physical activity for health. Individuals in rural areas were found to be more exposed to physical activity than urban residents. About 16% of respondents were

current khat chewers. Regarding injury, about 3% of respondents involved in a road traffic crash as a passenger, driver, or pedestrian during the past 12 months preceding the survey. The percentage of women respondents' age 30–49 years that had ever undergone screening for cervical cancer was 2.65%. (Table 1).

Table 1 Prevalence of behavioural risk factors by sex, Ethiopia STEPS 2015.

Behavioural risk factors	Males	Females	Total
Percentage who currently smoke tobacco	7.3% (6.1-8.6)	0.4% (0.3-0.6)	4.2% (3.5-4.9)
Percentage who currently smoke tobacco daily	6.2% (5.0-7.4)	0.2% (0.1-0.4)	3.5% (2.8-4.1)
Average age started smoking (years)	20.9 (19.8-22.0)	22.8 (19.1-26.5)	21.0 (19.9-22.0)
Percentage of daily smokers smoking manufactured cigarettes	91.5% (87.4-95.5)	48.4% (26.5-70.3)	89.4% (85.3-93.6)
Mean number of manufactured cigarettes smoked per day (by smokers of manufactured cigarettes)	7.3 (6.2-8.3)	2.4 (1.1-3.7)	7.1 (6.1-8.1)
Percentage who were lifetime abstainers from alcohol	45.1 (41.4-48.8)	57.3 (53.8-60.9)	50.7 (47.3-54.0)
Percentage who are past 12 month abstainers among alcohol consumers	5.1 (3.9-6.3)	5.2 (3.9-6.5)	5.1 (4.0-6.2)
Percentage who currently drink (drank alcohol in the past 30 days)	46.6 (43.0-50.2)	33.5 (30.3-36.7)	40.7 (37.4-43.9)
Percentage who engage in heavy episodic drinking (6 or more drinks on any occasion in the past 30 days)	20.5 (18.2-22.7)	2.7 (2.0-3.3)	12.4 (11.0-13.7)
Percentage who currently chew Khat	21.1 (17.6-24.7)	9.4 (7.2-11.5	15.8 (13.1-18.5)
Among the chewers during the past 12 months, percentage of respondents who chew Khat daily.	61.4 (53.9-68.8)	50.4 (41.7-59.2)	58.4 (51.6-65.2)
Mean number of days fruit consumed in a typical week	0.9 Days(0.8-1.0)	1.0days (0.9-1.1)	0.9 (0.8-1.0)
Mean number of servings of fruit consumed on average per day	0.3 (0.3-0.4)	0.3 (0.3-0.4)	0.3 (0.3-0.4)
Mean number of days vegetables consumed in a typical week	1.4 Days (1.3-1.6)	1.5 Days (1.4-1.7)	1.5 (1.3-1.6)
Mean number of servings of vegetables consumed on average per day	0.5 (0.4-0.6)	0.6 (0.5-0.8)	0.6 (0.5-0.7)
Percentage who ate less than 5 servings of fruit and/or vegetables on average per day	98%	97.1%	97.6%
Percentage who always or often add salt or salty sauce to their food before eating or while they are eating	60.0 % (55.9-64.1)	60.9% (57.0-64.8)	60.4% (56.6-64.2)
Percentage who always or often eat processed foods high in salt	9.8% (7.9-11.7)	8.3% (6.7-10.0)	9.1% (7.6-10.7)
Percentage with insufficient physical activity (defined as < 150 minutes of moderate-intensity activity per week, or equivalent)	4.0% (3.0-5.0)	7.9% (6.7-9.1)	5.8% (5.0-6.6)
Median time spent in physical activity on average per day (minutes)(presented with inter-quartile range)	300.0 Min (154.2-450.8)	188.6Min (71.4-360.0)	257.1Min (102.9-411.4)
Percentage not engaging in vigorous activity	22.1% (19.8-24.5)	66.7% (63.8-69.6)	42.5% (40.3-44.6)
Percent Involved in road traffic crashes in the past 12 month	3.3 (2.2-4.4)	1.9 (1.1-2.6)	2.7 (1.8-3.5)
Percentage of women age 30-49 years who have ever had a screening test for cervical cancer		2.65 (1.61 – 3.69)	

1Khat is a plant native to the Horn of Africa and the Arabian Peninsula. Khat chewing in Ethiopia is a social custom that dates back thousands of years. However, Khat is a strong stimulant that causes mild to moderate psychological dependence, although not as strong as that of alcohol and tobacco, and its consumption can have serious health and economic consequences.

2A 10-year CVD risk of ≥30% is defined according to age, sex, blood pressure, smoking status (current smokers or those who quit smoking less than 1 year before the assessment), total cholesterol, and diabetes (previously diagnosed or a fasting plasma glucose concentration>126 Mg/dl.

Physical Measurements-STEP 2

Prevalence of raised blood pressure (SBP > 140 and/or DBP > 90 mmHg) among Ethiopian adult population was 15.6%, with no difference by sex. Six percent of study participants had elevated blood glucose. Mean body mass index (BMI) was 20.4 (20.1 for men and 20.7 for women). Few individuals (6.3%) were overweight or obese, with a higher prevalence of overweight in urban residents.

Table 2 Mean body mass index and prevalence of raised blood pressure by sex, Ethiopia STEPS 2015.

Physical Measurements	Both Sexes	Males	Females
**Step 2 Physical Measurements**			
Mean body mass index - BMI (kg/m2)	20.4 (20.2 – 20.5)	20.1 (19.9 – 20.2)	20.7 (20.6 – 20.9)
Percentage who are overweight (BMI ≥ 25 kg/m2)	6.3 (5.4 –7.3)	4.4 (3.4 – 5.4)	8.8(7.6 – 10.0)
Percentage who are obese (BMI ≥ 30 kg/m2)	1.2(0.9 – 1.4)	0.5(0.2 – 0.8)	2.0(1.5 – 2.4)
Average waist circumference (cm)	NA	74.4 (73.9 – 75.0)	73.9 (73.3 – 74.4)
Mean systolic blood pressure - SBP (mmHg), including those currently on medication for raised BP	119.5 (118.8 – 120.2)	120.2 (119.2 – 121.1)	118.7 (117.9 – 119.5)
Mean diastolic blood pressure - DBP (mmHg), including those currently on medication for raised BP	77.5 (77.0 – 78.1)	76.5 (75.8 – 77.2)	78.8 (78.3 – 79.3)
Percentage with raised BP (SBP ≥ 140 and/or DBP ≥ 90 mmHg or currently on medication for raised BP)	16(14.8 – 17.3)	15.7 (13.9 – 17.5)	16.5 (15.0 – 17.9)
Percentage with raised BP (SBP ≥ 140 and/or DBP ≥ 90 mmHg) who are not currently on medication for raised BP	15.6 (14.4 – 16.9)	15.3 (13.5 – 17.1)	16.0 (14.6 – 17.5)

Biochemical Measurements – STEP 3

On average, 5.4 % of the study population had impaired fasting glycaemia and 5.6 % of the population has a blood cholesterol level of ≥ 190mg/dl. Only 1.6% of the study population was found to be free of any established NCD risk factors. Hence, a massive 98.4% has at least one risk factor. This indicates that the burden of NCDs is likely to become unbearable in the future in Ethiopia that needs government attention. Based on a number of risk factors such as smoking status, raised blood pressure, elevated blood glucose and raised total cholesterol, the proportion of 40–69 year old adults with a 10-year risk of cardiovascular disease ≥30% was also substantial at 4.7%.

Table 3 Mean body mass index and prevalence of raised blood pressure by sex, Ethiopia STEPS 2015

Variables	Both Sexes	Males	Females
**Step 3 Biochemical Measurement**			
Mean fasting blood glucose, including those currently on medication for elevated blood glucose in mg/dl	79.5 (78.3 – 80.7)	79.0 (77.6 – 80.4)	80.2 (79.0 – 81.3)
Percentage with impaired fasting glycaemia as defined below<br>· capillary whole blood value ≥ 100 mg/dl and <110 mg/dl	5.4(4.4 – 6.3)	5.1(3.9 – 6.3)	5.7(4.9 – 6.8)
Percentage with elevated fasting blood glucose as defined below or currently on medication for elevated blood glucose<br>· capillary whole blood value ≥ 110 mg/dl	5.9(4.9 – 6.9)	6.0(4.7 – 7.2)	5.8 (4.6 – 7.0)
Mean total blood cholesterol, including those currently on medication for raised cholesterol in mg/dl	130.9 (129.3 – 132.6)	124.4 (122.6 – 126.1)	139.2 (137.0 – 141.3)
Percentage with raised total cholesterol (≥ 190 mg/dl or currently on medication for raised cholesterol)	5.6 (4.8 – 6.4)	3.5 (2.7 – 4.3)	8.3 (7.1 – 9.4)
Mean salt intake (g/day	8.3 (8.2 – 8.4)	9.0 (8.9-9.1)	7.4 (7.3 – 7.4)
Cardiovascular disease (CVD) risk			
Percentage age 40-69 years with a 10-year CVD risk ≥ 30%, or with existing CVD2	4.7 (3.5-5.8)	3.7 (2.4-5.0)	6.0 (4.3-7.7)
**Summary of combined risk factors**			
• current daily smokers<br>• over-weight (BMI ≥ 25 kg/m2)<br>• less than 5 servings of fruits & vegetables per day<br>• raised BP (SBP ≥ 140 and/or DBP ≥ 90 mmHg or<br>• insufficient physical activity<br>• currently on medication for raised BP)			
Percentage with none of the above risk factors	1.6 (0.9-2.3)	1.2 (0.5-1.9)	2.1 (1.3-2.9)
Percentage with 1-2 of the above risk factors, age 15 to 44 years	95.0 (94.0-95.9)	95.3 (94.2-96.5)	94.5 (93.4-95.6)
Percentage with 1-2 of the above risk factors, age 45 to 69 years	89.6 (87.7-91.4)	91.0 (88.6-93.5)	87.3 (85.0-89.7)
Percentage with 1-2 of the above risk factors, age 15 to 69 years	94.0 (93.0-94.9)	94.5 (93.3-95.7)	93.3 (92.2-94.4)

Among modifiable risk factors, place of residence and physical inactivity were significantly associated ($p<0.001$) with raised blood pressure (SBP>=140 and/or DBP>=90); while other demographic and behavioural risk factors like age of respondents, ever consumed alcohol, and adding salt to food were also significantly associated ($p<0.05$) in bivariate analysis. Among the modifiable risk factors living in rural areas(41%), those who didn't consume alcohol(13%), and those who add salt sometime or never in their diet(13%) had a decreased risk of developing raised BP (SBP>=140 and/or DBP>=90). Likewise, among the modifiable risk factors, place of residence, consuming alcohol, adding salt to food, and raised BP were significantly associated ($p<0.001$) with raised blood glucose.

Based on the findings of multivariate logistic regression analysis, living in rural areas (30%), those who didn't consume alcohol (60%), and those who add salt sometime or never in their diet (41%), those who didn't chew khat (24%), and those who don't have raised blood pressure (40%) had a decreased risk of developing elevated blood glucose (a capillary whole blood value >=110mg/dl or currently on medication for DM.

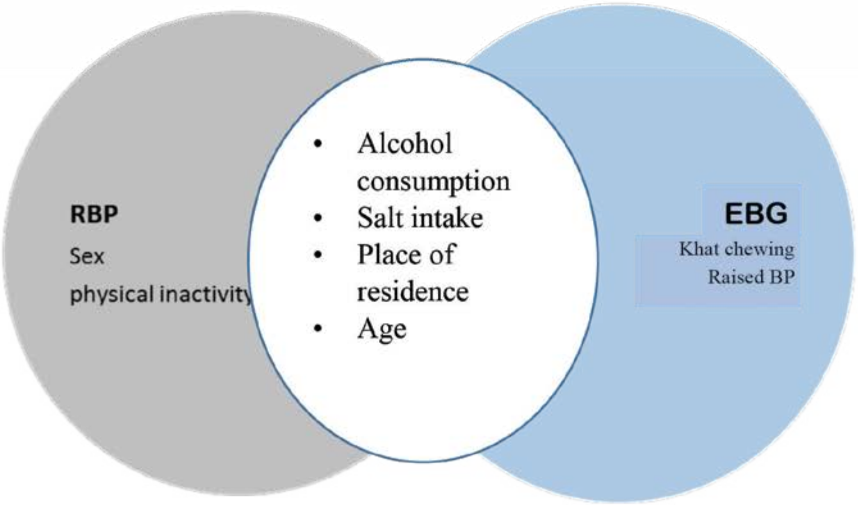

Fig 1 Shared and individual risk factors for both Raised Blood Pressure (RBP) and Elevated Blood Glucose (EBG), Ethiopia NCDsteps , 2015

Conclusions and Recommendations

The Ethiopia STEPs is the first nationally representative survey to collect comprehensive information on risk factors for NCDs, Injuries and violence in Ethiopia. It provides essential information on Key NCD indicators by age group, sex and residence, and region in some cases. This national survey has found the magnitude of the major NCD risk factors like behavioural risk factors (tobacco use, alcohol consumption, low fruit and vegetable consumption, khat consumption, and physical inactivity) and biological risk factors (overweight, obesity, raised blood pressure, elevated blood glucose and abnormal lipids). Most of the behavioural risk factors, such as tobacco use, alcohol consumption, khat consumption, were more prevalent among men. However, the biological risk factors, such as obesity, impaired fasting glycaemia, and raised total cholesterol were more prevalent among women.

In the present survey, 95% of the study participants were with 1-2 NCD risk factors and a prediction of the future disease burden prevailing in the population. The findings are useful in informing public health policy and the following recommendations are proposed:

Policy makers

- Revise and implement the existing NCD prevention and control strategy immediately to address the burden of NCD risk factors in Ethiopia. .In actions under this prevention and control strategy coordinate and collaborate with non-health sectors such as education, agriculture, broadcasting agency and involve other development partners.
- As indicated in the health sector transformation plan, there should be a mechanism to strengthen the issue of NCDs and their risk factors, including ensuring access to primary health care services for the early detection of biological risk factors and the promotion of healthy behaviours.
- Effectively implement the Framework Convention on Tobacco Control (FCTC) as well as the Tobacco Control Act and policy together with strong monitoring mechanisms.
- Adopt a national response to restrict distribution and use of selected NCD risk factors such as khat, alcohol and processed foods and fats and to promote fruit and vegetable consumption from lessons learned of the FCTC
- There should be an enhanced supportive mechanism from development partners to tackle the current burden of NCD risk factors.

Programme managers

- Re-orientate the primary health care system towards the early detection and treatment of hypertension and diabetes.
- Design and implement special and innovative behaviour change communication strategies tailored to different demographic groups in order to create public awareness to promote healthy behaviours and reduce risk factors.
- Integrate NCD prevention programmes in primary health care units with other health care programmes and ensure access to the community.
- Strengthen behavioural change and communication to inform the public about NCDs and their risk factors, bring awareness on the health risks associated with smoking and smoke cessation, drinking, benefits of eating fruits and/or vegetables, physical exercise, avoiding chewing khat, and regular visit for normal medical check-ups.
- Formulate strategies to promote the accessibility, availability and consumption of fruit and vegetables by all people.
- Strengthen and support programs preventing youth from engaging in substance use and abuse, including alcohol.

Researchers

- Assess interventions to promote healthy behaviours and reduce the burden of NCD risk factors in order to provide evidence generated locally for the implementation of NCD prevention and control activities by policy makers and programme managers.

- Evaluate the effectiveness of programmes implemented to prevent and control NCDs and NCD risk factors.
- Conduct specific NCD risk factors survey in detail to get in-depth information for specific behavioural risk factors.
- Disseminate and utilize findings of survey to inform non-communicable disease related program planning and actions.
- Strengthening community-based risk factor surveillance system and utilizing collected morbidity and mortality data through the existing system that lead to in depth analysis for follow up and future action.
- Conduct further analyses of the data, such as exploring relation of different characteristic of the population with determinant factors like socio-demographic, behavioural, and bi-chemical measurements.
- Related surveillance and pocket area studies are highly recommended to see the overall risk behaviours of younger age groups, including institution and areas where these age groups gather and get the exposure by exercising risky behaviour.
- Strengthening the capacity of health facilities enable them to offer the services related to NCDs and ensure that the health system adequately monitors compliance with national standards.

Acknowledgements

The Ethiopia non-communicable diseases (NCDs) Steps survey demonstrates the combined efforts of the Federal Ministry of Health, development partners, professional associations, and individuals without which this report could not have been possible.

The Ethiopian Public Health Institute would like to thank the following individuals/organization for their sterling support and contribution:

- All health development partners and collaborators who have provided support for the successful completion of this survey especially technical and financial support provided by WHO and assistance in procurement of reagent from abroad by UNICEF.
- Members of the Technical Working Group from respective organizations and professional associations who worked hard in providing technical support by reviewing and giving feedback on the overall process of the survey.
- The overall coordination and guidance provided by Health System Research Directorate of EPHI is also highly appreciated. Special thanks also go to the TB and HIV research directorate team who performed laboratory analyses for biological samples to make the report a reality. The institute would also like to extend its sincere gratitude to the survey participants who cooperated and participated in the realization of the survey.
- Last but not the least, the institute is grateful to all the field staff involved in data collection and supervision for their crucial roles played in achieving the survey goal.

List of Contributors

Name	Affiliation
Abebe Bekele	EPHI
Kassahun Amenu	EPHI
Theodros Getachew	EPHI
Atkure Defar	EPHI
Habtamu Teklie	EPHI
Terefe Gelibo	EPHI
Mekonnen Tadesse	EPHI
Dr. Alemayehu Bekele	EPHA
Prof. Yeweyenhareg Feleke	AAU/EMA
Dr. Asmamaw Bezabeh	WHO
Dr. Mussie G/Michael	FMOH
Feyissa Challa	EPHI
Tefera Tadele	EPHI
Abebech Asmamaw	EPHI
Dr. Yabetse Girma	EPHI
Tsehay Assefa	EPHI
Mulugeta Guta	EPHI
Kissi Mudie	EPHI
Dr. Yewondwossen Tadesse	AAU
Dr. Dejuma Yadeta	AAU
Dr. Tedla Kebede	AAU
Dr. Yoseph Mamo	JU
Dr. Solomon Teferra	AAU
Dr. Yibeltal Assefa	EPHI
Dr. Amha Kebede	EPHI
