# Supplementary material for: Prevalence of high bloodpressure, hyperglycemia, dyslipidemia, metabolic syndrome and their determinants in Ethiopia: Evidences from the National NCDs STEPS Survey, 2015
Source: PLoS One. 2018 May 9;13(5):e0194819. doi: 10.1371/journal.pone.0194819 (PMC5942803; doi:10.1371/journal.pone.0194819)
Supplement: S2 Appendix — (DOC) [file pone.0194819.s002.doc]

Ethiopian Public Health Institute

National HIV Reference Laboratory

**
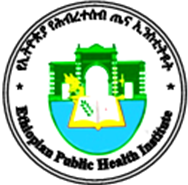
**

**Procedure for Performing Triglycerides**

| Compiler: | Signature: |
| --- | --- |
| Feyissa Challa |  |
| Zeleke Geto |  |
| Approver: Kissi Mudie | Signature: |
| Effective Date: 01,January 2016 | Version No:1.0 |
| Document No: NHIVRL/CCH/SOP5.5-010 | Copy No: |

REVISION AND AMENDMENT

Version Change History and Description of Amendment

| Revision No | Version No | Page No | Description of Amendment | Amendment  Date | Effective Date | Name & Signature of Reviewer | Name & Signature of approval |
| --- | --- | --- | --- | --- | --- | --- | --- |
|  |  |  |  |  |  |  |  |
|  |  |  |  |  |  |  |  |
|  |  |  |  |  |  |  |  |
|  |  |  |  |  |  |  |  |
|  |  |  |  |  |  |  |  |
|  |  |  |  |  |  |  |  |
|  |  |  |  |  |  |  |  |
|  |  |  |  |  |  |  |  |
|  |  |  |  |  |  |  |  |

| **Purpose** | This procedure provides instructions for performing in vitro test for the quantitative determination of the Triglycerides concentration in human serum and plasma on COBAS INTEGRA 400 plus and 501 systems |
| --- | --- |

| **Abbreviations** | | - TRIGL = Triglycerides - RT= Room Temperature - c.f.a.s. calibrator for automated system - EDTA-K3 ­= Potassium Ethyl Diamine Tetra Acetic Acid - NHIVRL = National HIV Reference Laboratory | | --- | |
| --- | --- | --- |

| **Clinical Utility** | Triglycerides are esters of the trihydric alcohol glycerol with 3 long-chain fatty acids. They are partly synthesized in the liver and partly ingested in food. The determination of triglycerides is utilized in the diagnosis and treatment of patients having diabetes mellitus, nephrosis, liver obstruction, lipid metabolism disorders and numerous other endocrine diseases. |
| --- | --- |

| **Principle** | Enzymatic colorimetric test |
| --- | --- |

| **Materials** | | **Reagents** | | --- | | 1. TRIG Cassettes 2. Sodium hydroxide   3. Activator  4. Deproteinizer  5. Cleaner |   **Reagents preparation:** Ready for use.  **Reagents stability and storage:**  Unopened at 2-8ºC up to the stated expiry date on cobas C pack lable  On board in use and refrigerated on the analyzer 8 weeks  **Note:** Store reagent kit upright   | **Supplies** | | --- | | 1. Sample Cup | | 1. Activator bottle | | 3. Waste Container | | 4. Cleaner Cassette | | 5. Micro Cuvettes | | **Equipment** | | - Cobas Integra 400 & 501 plus analyzer - Volumetric pipettes - Shaker - Centrifuge    Sample rack | |
| --- | --- | --- | --- | --- | --- | --- | --- | --- | --- | --- | --- |

| **Sample** | | **Sample type** | **Amount required** | **Transport and Storage** | **Stability** | | --- | --- | --- | --- | | Serum/ Plasma Li‑heparin | 300µl | Transport whole blood at RT Separate serum within 1 Hr.   store serum at 2-8⁰C |  at 4 °C for 10 -15 days |   ***Note****: -* Freeze only once.  **Limitations:**  Icterus: No significant interference up to an index of10 for conjugated bilirubin and 35  For unconjugated bilirubin (approximate conjugated bilirubin concentration: 171 μmol/L  Or 10 mg/dL; approximate unconjugated bilirubin concentration: 599 μmol/L or 35 mg/dL).  Hemolysis: No significant interference up to an H index of 700 (approximate hemoglobin concentration: 434 μmol/L or 700 mg/dL).  Lipemia: The L index correlates with sample turbidity but not with triglycerides level.  Extremely lipemic samples (triglycerides greater than 3000 mg/dL) can produce  Normal results  **Sample retention:** Serum/Plasma is discarded after 1 week |
| --- | --- | --- | --- | --- | --- | --- | --- | --- | --- |

| **Special Safety Precautions** | RRefer to the NHIVRL Safety Guidelines for standard safety procedures |
| --- | --- |

| **Calibration** | | **Calibrator** | **Level** | **Stability** | **Frequency** | **Preparation (y/n)** | | --- | --- | --- | --- | --- | |  c.f.a.s   Use deionized water as zero calibrator | Lavel 1  Lavel 2 | *Unopened at 2-8ºC up to the stated expiry date  Stability of the components in the reconstituted calibrator:  at 15‑25 °C 8 hours  at 2‑8 °C 2 days |  After reagent lot change  When Quality control results indicate need for recalibration. | Yes |   **Calibrator preparation:**  Carefully open one bottle avoiding the loss of lyophilizate, and pipette in exactly 3.0 mL of distilled/deionized water. Carefully close the bottle and dissolve the contents completely by occasional gentle swirling within 30 minutes. Avoid the formation of foam  **Note:**   - Calibrators must be at room temperature before use - Write the reconstitution date on the bottles. |
| --- | --- | --- | --- | --- | --- | --- | --- | --- | --- | --- | --- |

| **Quality Control** | | Control | Level | Stability | Frequency | Preparation Y/N | | --- | --- | --- | --- | --- | | PreciControl ClinChem Multi 1  PreciControl ClinChem Multi 2 | PCCC1  PCCC2 | Stability of components after reconstitution:  at 15-25 °C for 12 hours  at 2-8 °C for 5 days | *When the test performed.   - After Calibration | Yes |     **Control preparation:**  Carefully open one bottle, avoiding the loss of lyophilizate, and pipette in exactly 5.0 mL of distilled/deionizer water. Carefully close the bottle and dissolve the contents completely by occasional gentle swirling within 30 minutes. Avoid the formation of foam  **Note:**   - Controls must be kept at room temperature before use - Controls must be within range. If out of range, repeat the run. If still out of the range. Investigate for root cause. (Reagent, Calibration and QC Preparation….etc ) - Solve the Problem, Document and rerun the Quality controls |
| --- | --- | --- | --- | --- | --- | --- | --- | --- | --- | --- | --- |

| **Procedure** | Refer to cobas-integra 400 & 501 manual | |
| --- | --- | --- |
| **Result Interpretation** | | | **Step** | **Action** | | --- | --- | | 1 | Measuring range  0.1‑10.0 mmol/L (8.85‑885 mg/dL)  Determine samples having higher concentrations via the rerun function.  Dilution of samples via the rerun function is a 1:5 dilution. Results from  Samples diluted using the rerun function are automatically multiplied by  A factor of 5.  Lower detection limit of the test is 8.85 mg/dL  The detection limit represents the lowest measurable analyte level that can be distinguished from zero. It is calculated as the value lying three standard deviations above that of a zero sample | | 2 | Determine samples having higher concentrations via the rerun function. Dilution of samples via the rerun function is a 1:10 dilution. Results from samples diluted by the rerun function are automatically multiplied by a factor of 10. If manual dilution, then multiply the result by the dilution factor. | |
| Critical Value | | Not applicable  Expected value : Expectes values |

| **Limitations** | Icterus: No significant interference up to an I index of 10 for conjugated bilirubin And 35 for unconjugated bilirubin (approximate conjugated bilirubin concentration:  171 μmol/L or 10 mg/dL; approximate unconjugated bilirubin concentration: 599 μmol/L or 35 mg/dL).  Hemolysis: No significant interference up to an H index of 700 (approximate Hemoglobin concentration: 434 μmol/L or 700 mg/dL).  Lipemia: The L index correlates with sample turbidity but not with triglycerides level. Extremely lipemic samples (triglycerides greater than 3000 mg/dL) can produce normal results.  . |
| --- | --- |
| Performance Characteristics | Precision was determined using human samples and controls in an internal protocol. Repeatabilityb (n = 20), intermediate precisionc (2 aliquots per run, 2 runs per day, 20 days). The following results were obtained: |

| **Subsidiary**   | Document   | Document Unique ID | Document Name | | --- | --- | | NHIVRL/ALS/QPM/4.2/001 | Quality Policy Manual | |  |  | | --- | --- | --- | --- | --- | --- | --- | |  |  | |
| --- | --- | --- | --- | --- | --- | --- | --- | --- | --- |
|
|

| **Reference** | - Roche Cobas Integra 400 & 501 plus Operator Manual - Roche Cobas Integra 400 & 501 plus Package Inserts - Tietz NW, editor. Text book of Clinical Chemistry.3rd ed. Philadelphia:   WB Saunders,2001   - Lothar Thomas: Clinical Laboratory Diagnostics, use and assessment of Clinical Laboratory Results 1st ed.1998, |
| --- | --- |

Declaration

I, the undersigned laboratory personnel, certify that I am conducting every steps of the procedures incorporated in this SOP after a prior reading.

Name Signature and Date

**……………………………... ……………………………...**

**……………………………... ……………………………...**

**……………………………... ……………………………...**

**……………………………... ……………………………...**

**……………………………... ……………………………...**

**……………………………... ……………………………...**

**……………………………... ……………………………...**

**……………………………... ……………………………...**

**……………………………... ……………………………...**

**……………………………... ……………………………...**

**……………………………... ……………………………...**
