# Supplementary material for: Prevalence of high bloodpressure, hyperglycemia, dyslipidemia, metabolic syndrome and their determinants in Ethiopia: Evidences from the National NCDs STEPS Survey, 2015
Source: PLoS One. 2018 May 9;13(5):e0194819. doi: 10.1371/journal.pone.0194819 (PMC5942803; doi:10.1371/journal.pone.0194819)
Supplement: S3 Appendix — (PDF) [file pone.0194819.s003.pdf]

Participant Identification Number └─┴─┴─┴─┴─┘

Ethiopia STEPS survey questionnaire on Risk Factors for chronic Non-Communicable Diseases and Prevalence of selected NCDs, 2015

The Ethiopian Public Health Institute

Survey Information

Location and Date	Response	Code
Enumeration area ID	└─┴─┴─┘	I1
Region [2 digits]	└─┴─┘	**I1-B**
Woreda [text]		**X1**
Interviewer ID	└─┴─┘	I3
Date of completion of the instrument	└─┴─┘ └─┴─┘ └─┴─┴─┴─┘<br>dd mm year	I4

**1 =Tigray; 2=Afar; 3=Amhara; 4=Oromia, 5=Somali; 6= B-gumuz; 7= SNNP; 8=Gambella; 9=Harari 10= Dire Dawa; 11= Addis Ababa*

Consent, Interview Language and Name	Response	Code
Consent has been read and obtained	Yes 1<br>No 2 **If NO, END**	I5
Interview Language	English 1<br>*Amharic* 2<br>*Oromifa* 3<br>*Tigrigna* 4<br>Others Specify 5________________________	I6
Start Ttime of interview<br>(24 hour clock)	└─┴─┘ : └─┴─┘<br>hrs mins	I7
Full Name of study participant (Grand Father, First and Middle Name)	______________________________	I8-I9
**Additional Information that may be helpful**		
Contact phone number where possible		I10

Participant Identification Number └─┴─┴─┴─┴─┘

Step 1 Demographic Information

**CORE: Demographic Information**		
**Question**	**Response**	**Code**
Sex (*Record Male / Female as observed*)	Male 1<br>Female 2	C1
How old are you?	Years └─┴─┘	C3
In total, how many years have you spent at school and in full-time study (excluding pre-school)?	Years └─┴─┘	C4

**EXPANDED: Demographic Information**		
What is the **highest level of education** you have completed?	No formal schooling 1<br>Less than primary school 2<br>Primary school completed 3<br>Secondary school completed 4<br>College/University completed 6<br>Post graduate degree 7<br>Refused 88	C5
What is your ethnic **background**?	Oromo 1<br>Amhara 2<br>Tigray 3<br>Somali 4<br>Wolayita 5<br>Sidama 6<br>Guragie 7<br>Hadiya 8<br>Afar 9<br>Gamo 10<br>Others 11 Sp____________________<br>Refused 88	C6
What is your **marital status**?	Never married 1<br>Currently married 2<br>Separated 3<br>Divorced 4<br>Widowed 5<br>Cohabitating 6<br>Refused 88	C7
Which of the following best describes your **main work** status over the past 12 months?	Government employee 1<br>Non-government Employee 2<br>Private employee 3<br>Private Skilled worker 4<br>Farmer 5<br>Trader 6<br>Student 7<br>Homemaker\housewife 8<br>Retired 9<br>Unemployed (able to work) 10<br>Unemployed (unable to work) 11<br>Others 12 ______________________	C8

	Refused 88	
How many people older than 18 years, including yourself (if older than 18 years), live in your household?	Number of people └─┴─┘	C9

**Question**	**Response**		**Code**
Taking **the past year**, can you tell me what the average earnings (Birr) of the household have been?<br>*(RECORD ONLY ONE, NOT ALL 3)*	Per week └─┴─┴─┴─┴─┴─┴─┴─┘	*Go to T1*	C10a
	OR per month └─┴─┴─┴─┴─┴─┴─┴─┘	*Go to T1*	C10b
	OR per year └─┴─┴─┴─┴─┴─┴─┴─┘	*Go to T1*	C10c
	Refused 88		C10d
If you don't know the amount, can you give an **estimate** of the annual household income if I read some options to you? Is it<br>*(READ OPTIONS)*	≤ 12,000 Birr	1	C11
	More than 12,000 ≤ 18,000 Birr	2	
	More than 18,000 ≤23,300	3	
	More than 23,300 ≤ 30,000	4	
	More than 30,000	5	
	Don't Know	77	
	Refused	88	

Participant Identification Number └─┴─┴─┴─┴─┘

Step 1 Behavioural Measurements

**CORE: Tobacco Use**		
Now I am going to ask you some questions about tobacco use.		
**Question**	**Response**	**Code**
Do you **currently** smoke any **tobacco** products, such as cigarettes, cigars or pipes, *gaya*? *(USE SHOWCARD)*	Yes 1<br>No 2 *If No, go to T8*	T1
Do you currently smoke tobacco products **daily**?	Yes 1<br>No 2	T2
How old were you when you **first started** smoking?	Age (years)<br>Don't know 77 └─┴─┘ *If Known, go to T5a/T5aw*	T3
Do you remember how long ago it was? *(RECORD ONLY 1, NOT ALL 3)* *Don't know 77*	In Years └─┴─┘ *If Known, go to T5a/T5aw*	T4a
	OR in Months └─┴─┘ *If Known, go to T5a/T5aw*	T4b
	OR in Weeks └─┴─┘	T4c
On average, **how many** of the following products do you smoke **each day/week?** *(IF LESS THAN DAILY, RECORD WEEKLY)* *(RECORD FOR EACH TYPE, USE SHOWCARD)* *Don't Know 7777*	DAILY↓ WEEKLY↓	
	Manufactured cigarettes └─┴─┴─┴─┘ └─┴─┴─┴─┘	T5a/T5aw
	Hand-rolled cigarettes └─┴─┴─┴─┘ └─┴─┴─┴─┘	T5b/T5bw
	Pipes full of tobacco └─┴─┴─┴─┘ └─┴─┴─┴─┘	T5c/T5cw
	Number of Shisha sessions └─┴─┴─┴─┘ └─┴─┴─┴─┘	T5e/T5ew
	Gaya └─┴─┴─┴─┘ └─┴─┴─┴─┘	T5X/ T5Xw
	Other └─┴─┴─┴─┘ └─┴─┴─┴─┘ *If Other, go to T5other, else go to T6*	T5f/T5fw
	Other (please specify): ______________________	T5other/ T5otherw
During the past 12 months, have you tried to **stop smoking**?	Yes 1<br>No 2	T6
During any visit to a doctor or other health worker in the past 12 months, were you advised to quit smoking tobacco?	Yes 1 *If T2=Yes, go to T12; if T2=No, go to T9*<br>No 2 *If T2=Yes, go to T12; if T2=No, go to T9*<br>No visit during the past 12 months 3 *If T2=Yes, go to T12; if T2=No, go to T9*	T7
In the past, did you **ever smoke** any tobacco products? *(USE SHOWCARD)*	Yes 1<br>No 2 *If No, go to T12*	T8
In the past, did you **ever** smoke **daily**?	Yes 1 *If T1=Yes, go to T12, else go to T10*<br>No 2 *If T1=Yes, go to T12, else go to T10*	T9
How old were you when you **stopped** smoking?	Age (years)<br>Don't Know 77 └─┴─┘ *If Known, go to T12*	T10
How **long ago** did you stop smoking? *(RECORD ONLY 1, NOT ALL 3)* *Don't Know 77*	Years ago └─┴─┘ *If Known, go to T12*	T11a
	OR Months ago └─┴─┘ *If Known, go to T12*	T11b
	OR Weeks ago └─┴─┘	T11c

Participant Identification Number └─┴─┴─┴─┴─┘

**CORE: Tobacco Use, cont.**			
**Question**	**Response**		**Code**
Do you **currently use** any **smokeless tobacco** products such as *snuff(Suret), chewing tobacco, (USE SHOWCARD)*	Yes 1<br>No 2	*If No, go to T15*	T12
Do you **currently use smokeless tobacco** products **daily?**	Yes 1<br>No 2	*If No, go to T14aw*	T13
On average, how many **times a day/week** do you use ….<br>*(IF LESS THAN DAILY, RECORD WEEKLY)*<br>*(RECORD FOR EACH TYPE, USE SHOWCARD)*<br>*Don't Know 7777*		DAILY↓ WEEKLY↓	
	Snuff, by mouth	└─┴─┴─┴─┘ └─┴─┴─┴─┘	T14a/ T14aw
	Snuff, by nose	└─┴─┴─┴─┘ └─┴─┴─┴─┘	T14b/ T14bw
	Chewing tobacco	└─┴─┴─┴─┘ └─┴─┴─┴─┘	T14c/ T14cw
	Other	└─┴─┴─┴─┘ └─┴─┴─┴─┘<br>*If Other, go to T14other, if T13=No, go to T16, else go to T17*	T14e/ T14ew
	Other (please specify):	____________________/<br>*f T13=No, go to T16, else go to T17*	T14other/ T14otherw
In the **past**, did you **ever use** smokeless tobacco products such as *snuff, chewing tobacco*?	Yes 1<br>No 2	*If No, go to T17*	T15
In the **past**, did you **ever use** smokeless tobacco products such as *snuff, chewing tobacco daily*?	Yes 1<br>No 2		T16
During the past 30 days, did someone smoke **in your home**?	Yes 1<br>No 2		T17
During the past 30 days, did someone smoke in closed areas **in your workplace** (in the building, in a work area or a specific office)?	Yes 1<br>No 2<br>Don't work in a closed area 3		T18

Participant Identification Number └─┴─┴─┴─┴─┘

**Tobacco Policy**		
You have been asked questions on tobacco consumption before. The next questions ask about tobacco control policies. They include questions on your exposure to the media and advertisement, on cigarette promotions, health warnings and cigarette purchases.		
**Question**	**Response**	**Code**
During the past 30 days, have you noticed information about the dangers of smoking cigarettes or that encourages quitting through the following media? *(RECORD FOR EACH)*		
Newspapers or magazines	Yes 1<br>No 2<br>Don't know 77	TP1a
Television	Yes 1<br>No 2<br>Don't know 77	TP1b
Radio	Yes 1<br>No 2<br>Don't know 77	TP1c
During the past 30 days, have you noticed any **advertisements** or **signs** promoting cigarettes in stores where cigarettes are sold?	Yes 1<br>No 2<br>Don't know 77	TP2
During the past 30 days, have you noticed any of the following types of cigarette promotions? *(RECORD FOR EACH)*		
Free samples of cigarettes	Yes 1<br>No 2<br>Don't know 77	TP3a
Cigarettes at sale prices	Yes 1<br>No 2<br>Don't know 77	TP3b
Coupons for cigarettes	Yes 1<br>No 2<br>Don't know 77	TP3c
Free gifts or special discount offers on other products when buying cigarettes	Yes 1<br>No 2<br>Don't know 77	TP3d
Clothing or other items with a cigarette brand name or logo	Yes 1<br>No 2<br>Don't know 77	TP3e
Cigarette promotions in the mail	Yes 1<br>No 2<br>Don't know 77	TP3f
The next questions TP4 – TP7 are administered to current smokers only.		
During the past 30 days, did you notice any **health warnings on cigarette packages**?	Yes 1<br>No 2 *If no, go to TP6*<br>Did not see any cigarette packages 3 *If "did not see any cigarette packages", go to TP6*<br>Don't know 77 *If Don't know, go to TP6*	TP4
During the past 30 days, have warning labels on cigarette packages led you to **think about quitting**?	Yes 1<br>No 2<br>Don't know 77	TP5
The last time you bought manufactured cigarettes for yourself, **how many cigarettes** did you buy in total?	Number of cigarettes └─┴─┴─┴─┴─┘<br>Don't know or Don't smoke or purchase manuf. cigarettes 7777 *If "Don't know or don't smoke or purchase manuf. cig.", end section*	TP6
In total, **how much money** did you pay for this purchase?	Amount Birr └─┴─┘ Cents └─┴─┘<br>Don't know 7777<br>Refused 8888	TP7

Participant Identification Number └─┴─┴─┴─┴─┘

CORE: Alcohol Consumption

The next questions ask about the consumption of alcohol.

Question	Response	Code
Have you **ever** consumed any alcohol such beer, Tella, Bordie, Tej, Arake, wine, spirits, beherawi, ye bale zaf? *(USE SHOWCARD OR SHOW EXAMPLES)*	Yes 1<br>No 2 *If No, go to A16*	A1
Have you consumed any alcohol within the **past 12 months**?	Yes 1 *If Yes, go to A4*<br>No 2	A2
Have you stopped drinking due to health reasons, such as a negative impact on your health or on the advice of your doctor or other health worker?	Yes 1 *If Yes, go to A16*<br>No 2 *If No, go to A16*	A3
During the past 12 months, **how frequently** have you had at least one standard alcoholic drink? *(READ RESPONSES, USE SHOWCARD)*	Daily 1<br>5-6 days per week 2<br>3-4 days per week 3<br>1-2 days per week 4<br>1-3 days per month 5<br>Less than once a month 6	A4
Have you consumed any alcohol within the **past 30 days**?	Yes 1<br>No 2 *If No, go to A13*	A5
During the past 30 days, on how many **occasions** did you have at least one standard alcoholic drink?	Number<br>Don't know 77 └─┴─┘	A6
During the past 30 days, when you drank alcohol, how many **standard drinks on average** did you have during one drinking occasion? *(USE SHOWCARD)*	Number<br>Don't know 77 └─┴─┘	A7
During the past 30 days, what was the **largest number** of standard drinks you had on a single occasion, counting all types of alcoholic drinks together?	Largest number<br>Don't Know 77 └─┴─┘	A8
During the past 30 days, how many times did you have **six or more** standard drinks in a single drinking occasion?	Number of times<br>Don't Know 77 └─┴─┘	A9
During the past 30 days, when you consumed an alcoholic drink, how often was it with meals? Please do not count snacks.	Usually with meals 1<br>Sometimes with meals 2<br>Rarely with meals 3<br>Never with meals 4	X6
During each of the **past 7 days**, how many standard drinks did you have each day? *(USE SHOWCARD)* *Don't Know 77*	Monday └─┴─┘	A10a
	Tuesday └─┴─┘	A10b
	Wednesday └─┴─┘	A10c
	Thursday └─┴─┘	A10d
	Friday └─┴─┘	A10e
	Saturday └─┴─┘	A10f
	Sunday └─┴─┘	A10g

**CORE: Alcohol Consumption, cont.**		
I have just asked you about your consumption of alcohol during the past 7 days. The questions were about alcohol in general, while the next questions refer to your consumption of homebrewed alcohol, alcohol brought over the border/from another country, any alcohol not intended for drinking or other untaxed alcohol. Please only think about these types of alcohol when answering the next questions.		
**Question**	**Response**	**Code**
During the **past 7 days**, did you consume any **homebrewed** alcohol, like Tella, Tej, Katikalla, Bordie? *(USE SHOWCARD)*	Yes 1 No 2 *If No, go to A13*	A11
On average, **how many standard drinks** of the following did you consume **during the past 7 days**? *(USE SHOWCARD)* *Don't Know 77*	Homebrewed spirits, e.g. Katikala └─┴─┘	A12a
	Homebrewed beer or wine, e.g. Tella, Tej └─┴─┘	A12b
	Alcohol brought over the border/from another country └─┴─┘	A12c
	Alcohol not intended for drinking, e.g. alcohol-based medicines, perfumes, after shaves └─┴─┘	A12d
	Other untaxed alcohol in the country └─┴─┘	A12e
During the **past 12 months,** how often have you found that you were not able to stop drinking once you had started?	Daily or almost daily 1 Weekly 2 Monthly 3 Less than monthly 4 Never 5	A13
During the **past 12 months,** how often have you failed to do what was normally expected from you because of drinking?	Daily or almost daily 1 Weekly 2 Monthly 3 Less than monthly 4 Never 5	A14
During the **past 12 months,** how often have you needed a first drink in the morning to get yourself going after a heavy drinking session?	Daily or almost daily 1 Weekly 2 Monthly 3 Less than monthly 4 Never 5	A15
During the **past 12 months**, have you had family problems or problems with your partner due to **someone else's** drinking?	Yes, more than monthly 1 Yes, monthly 2 Yes, several times but less than monthly 3 Yes, once or twice 4 No 5	A16

Diet

The next questions ask about the fruits and vegetables that you usually eat. I have a nutrition card here that shows you some examples of local fruits and vegetables. Each picture represents the size of a serving. As you answer these questions please think of a typical week in the last year.

Question	Response	Code
In a typical week, on how many days do you **eat fruit**? *(USE SHOWCARD)*	Number of days<br>Don't Know 77 └─┴─┘ *If Zero days, go to D3*	D1
How many **servings** of fruit do you eat on **one** of those days? *(USE SHOWCARD)*	Number of servings<br>Don't Know 77 └─┴─┘	D2
In a typical week, on how many days do you **eat vegetables**? *(USE SHOWCARD)*	Number of days<br>Don't Know 77 └─┴─┘ *If Zero days, go to D5*	D3
How many **servings** of vegetables do you eat on one of those days? *(USE SHOWCARD)*	Number of servings<br>Don't know 77 └─┴─┘	D4

Dietary salt

With the next questions, we would like to learn more about salt in your diet. Dietary salt includes ordinary table salt, unrefined salt such as sea salt, iodized salt, salty stock cubes and powders, and salty sauces such as soya sauce or fish sauce (see showcard). The following questions are on adding salt to the food right before you eat it, on how food is prepared in your home, on eating processed foods that are high in salt such as *[insert country specific examples]*, and questions on controlling your salt intake. Please answer the questions even if you consider yourself to eat a diet low in salt.

How often do you **add salt or a salty sauce such as soya sauce** to your food right before you eat it or as you are eating it?<br>*(SELECT ONLY ONE)*<br>*(USE SHOWCARD)*	Always 1<br>Often 2<br>Sometimes 3<br>Rarely 4<br>Never 5<br>Don't know 77	D5
How often is **salt, salty seasoning or a salty sauce added** in cooking or preparing foods in your household?	Always 1<br>Often 2<br>Sometimes 3<br>Rarely 4<br>Never 5<br>Don't know 77	D6
How often do you eat **processed food high in salt**? By processed food high in salt, I mean foods that have been altered from their natural state, such as packaged salty snacks, canned salty food including pickles and preserves, salty food prepared at a fast food restaurant, cheese, bacon, Mitmitta, and processed meat like Quantta<br>*(USE SHOWCARD)*	Always 1<br>Often 2<br>Sometimes 3<br>Rarely 4<br>Never 5<br>Don't know 77	D7
**How much salt or salty sauce** do you think you consume?	Far too much 1<br>Too much 2<br>Just the right amount 3<br>Too little 4<br>Far too little 5<br>Don't know 77	D8

Diet

Participant Identification Number |__|__|__|__|__|

Question	Response	Code
How important to you is **lowering the salt** in your diet?	Very important 1<br>Somewhat important 2<br>Not at all important 3<br>Don't know 77	D9
Do you think that too much salt or salty sauce in your diet could cause a **health problem**?	Yes 1<br>No 2<br>Don't know 77	D10
Do you do any of the following on a regular basis to **control your salt intake**?<br>*(RECORD FOR EACH)*		
Limit consumption of processed foods	Yes 1<br>No 2	D11a
Look at the salt or sodium content on food labels	Yes 1<br>No 2	D11b
Buy low salt/sodium alternatives	Yes 1<br>No 2	D11c
Use spices other than salt when cooking	Yes 1<br>No 2	D11d
Avoid eating foods prepared outside of a home	Yes 1<br>No 2	D11e
Do other things specifically to control your salt intake	Yes 1 *If Yes, go to D11other*<br>No 2	D11f
Other (please specify)		D11other
The next questions ask about the oil or fat that is most often used for meal preparation in your household, and about meals that you eat outside a home.		
What type of **oil or fat is most often** used for meal preparation in your household?<br><br>*(USE SHOWCARD)*<br>*(SELECT ONLY ONE)*	Vegetable oil 1<br>Homemade oil product 2<br>Butter 3<br>Margarine 4<br>Solid fats 8<br>Other 5 *If Other, go to D12 other*<br>None in particular 6<br>None used 7<br>Don't know 77	D12
	Other specify ________	D12other
On average, how many meals per week do you eat that were not prepared at a home? By meal, I mean breakfast, lunch and dinner.	Number<br>Don't know 77 |__|__|	D13

Participant Identification Number └─┴─┴─┴─┴─┘

CORE: Physical Activity

Next I am going to ask you about the time you spend doing different types of physical activity in a typical week. Please answer these questions even if you do not consider yourself to be a physically active person.
Think first about the time you spend doing work. Think of work as the things that you have to do such as paid or unpaid work, study/training, household chores, harvesting food/crops, fishing or hunting for food, seeking employment. In answering the following questions 'vigorous-intensity activities' are activities that require hard physical effort and cause large increases in breathing or heart rate, 'moderate-intensity activities' are activities that require moderate physical effort and cause small increases in breathing or heart rate.

Question	Response	Code
**Work**		
Does your work involve vigorous-intensity activity that causes large increases in breathing or heart rate like *carrying or lifting heavy loads, digging or construction work, cutting fire and other wood* for at least 10 minutes continuously? *(USE SHOWCARD)*	Yes 1<br>No 2 *If No, go to P 4*	P1
In a typical week, on how many days do you do vigorous-intensity activities as part of your work?	Number of days └─┘	P2
How much time do you spend doing vigorous-intensity activities at work on a typical day?	Hours : minutes └─┴─┘ : └─┴─┘ hrs mins	P3 (a-b)
Does your work involve/ or do you do moderate-intensity activity, that causes small increases in breathing or heart rate such as brisk walking *or carrying light loads, washing clothes* for at least 10 minutes continuously? *(USE SHOWCARD)*	Yes 1<br>No 2 *If No, go to P 7*	P4
In a typical week, on how many days do you do moderate-intensity activities as part of your work?	Number of days └─┘	P5
How much time do you spend doing moderate-intensity activities at work on a typical day?	Hours : minutes └─┴─┘ : └─┴─┘ hrs mins	P6 (a-b)
**Travel to and from places**		
The next questions exclude the physical activities at work that you have already mentioned.<br>Now I would like to ask you about the usual way you travel to and from places. For example to work, for shopping, to market, to place of worship, to place of meeting.		
Do you walk or use a bicycle *(pedal cycle)* for at least 10 minutes continuously to get to and from places?	Yes 1<br>No 2 *If No, go to P 10*	P7
In a typical week, on how many days do you walk or bicycle for at least 10 minutes continuously to get to and from places?	Number of days └─┘	P8
How much time do you spend walking or bicycling for travel on a typical day?	Hours : minutes └─┴─┘ : └─┴─┘ hrs mins	P9 (a-b)

Question	Response	Code
**Recreational activities**		
The next questions exclude the work and transport activities that you have already mentioned.<br>Now I would like to ask you about sports, fitness and recreational/leisure activities.		
Do you do any vigorous-intensity sports, fitness or recreational *(leisure)* activities that cause large increases in breathing or heart rate like *running or football, local dancing* for at least 10 minutes continuously? *(USE SHOWCARD)*	Yes 1<br>No 2 *If No, go to P 13*	P10
In a typical week, on how many days do you do vigorous-intensity sports, fitness or recreational *(leisure)* activities?	Number of days └─┘	P11
How much time do you spend doing vigorous-intensity sports, fitness or recreational activities on a typical day?	Hours : minutes └─┴─┘ : └─┴─┘ hrs mins	P12 (a-b)

**Physical Activity, Continued**		
**Question**	**Response**	**Code**
Do you do any moderate-intensity sports, fitness or recreational (leisure) activities that cause a small increase in breathing or heart rate such as brisk walking, cycling, swimming, volleyball for at least 10 minutes continuously? (USE SHOWCARD)	Yes 1<br>No 2 *If No, go to P16*	P13
In a typical week, on how many days do you do moderate-intensity sports, fitness or recreational (leisure) activities?	Number of days └─┘	P14
How much time do you spend doing moderate-intensity sports, fitness or recreational *(leisure)* activities on a typical day?	Hours : minutes └─┴─┘ : └─┴─┘ hrs mins	P15 (a-b)

**Physical Activity**		
**Sedentary behavior**		
The following question is about sitting or reclining at work, at home, getting to and from places, or with friends including time spent sitting at a desk, sitting with friends, traveling in car, bus, train, reading, playing games/cards or watching television, but do not include time spent sleeping. *(USE SHOWCARD)*		
How much time do you usually spend sitting or reclining on a typical day?	Hours : minutes └─┴─┘ : └─┴─┘ hrs mins	P16 (a-b)

Participant Identification Number └─┴─┴─┴─┴─┘

History of Raised Blood Pressure

Question	Response	Code
Have you ever had your blood pressure measured by a doctor or other health worker?	Yes 1<br>No 2 *If No, go to X10*	H1
Have you ever been told by a doctor or other health worker that you have raised blood pressure or hypertension?	Yes 1<br>No 2 *If No, go to X10*	H2a
Have you been told in the past 12 months?	Yes 1<br>No 2	H2b
In the past two weeks, have you taken any drugs (medication) for raised blood pressure prescribed by a doctor or other health worker?	Yes 1<br>No 2	H3
Have you ever seen a traditional healer for raised blood pressure or hypertension?	Yes 1<br>No 2	H4
Are you currently taking any herbal or traditional remedy for your raised blood pressure?	Yes 1<br>No 2	H5
Has any of your family members (biological parents, siblings or children) ever had raised blood pressure or hypertension?	Yes 1<br>No 2	X10

History of Diabetes

Question	Response	Code
Have you ever had your blood sugar measured by a doctor or other health worker?	Yes 1<br>No 2 *If No, go to X11*	H6
Have you ever been told by a doctor or other health worker that you have raised blood sugar or diabetes?	Yes 1<br>No 2 *If No, go to X11*	H7a
Have you been told in the past 12 months?	Yes 1<br>No 2	H7b
In the past two weeks, have you taken any drugs (medication) for diabetes prescribed by a doctor or other health worker?	Yes 1<br>No 2	H8
Are you currently taking insulin for diabetes prescribed by a doctor or other health worker?	Yes 1<br>No 2	H9
Have you ever seen a traditional healer for diabetes or raised blood sugar?	Yes 1<br>No 2	H10
Are you currently taking any herbal or traditional remedy for your diabetes?	Yes 1<br>No 2	H11
Has any of your family members (biological parents, siblings or children) ever had raised blood sugar or Diabetes?	Yes 1<br>No 2	X11

Participant Identification Number └─┴─┴─┴─┴─┘

**History of Raised Total Cholesterol**		
**Question**	**Response**	**Code**
Have you ever had your cholesterol (fat levels in your blood) measured by a doctor or other health worker?	Yes 1<br>No 2 *If No, go to X12*	H12
Have you ever been told by a doctor or other health worker that you have raised cholesterol?	Yes 1<br>No 2 *If No, go to X12*	H13a
Have you been told in the past 12 months?	Yes 1<br>No 2	H13b
In the past two weeks, have you taken any oral treatment (medication) for raised total cholesterol prescribed by a doctor or other health worker?	Yes 1<br>No 2	H14
Have you ever seen a traditional healer for raised cholesterol?	Yes 1<br>No 2	H15
Are you currently taking any herbal or traditional remedy for your raised cholesterol?	Yes 1<br>No 2	H16
Has any of your family members (biological parents, siblings or children) ever had raised Cholesterol?	Yes 1<br>No 2	X12

**History of Cardiovascular Diseases**		
Have you ever had a heart attack or chest pain from heart disease (angina) or a stroke (cerebrovascular accident or incident)?	Yes 1<br>No 2	H17
Are you currently taking aspirin regularly to prevent or treat heart disease?	Yes 1	H18
Are you currently taking statins (Lovastatin/Simvastatin/Atorvastatin or any other statin) regularly to prevent or treat heart disease?	Yes 1<br>No 2	H19

**Lifestyle Advice**		
During the past three years, has a doctor or other health worker advised you to do any of the following? *(RECORD FOR EACH)*		
Quit using tobacco or don't start	Yes 1<br>No 2	H20a
Reduce salt in your diet	Yes 1<br>No 2	H20b
Eat at least five servings of fruit and/or vegetables each day	Yes 1<br>No 2	H20c
Reduce fat in your diet	Yes 1<br>No 2	H20d
Start or do more physical activity	Yes 1<br>No 2	H20e
Maintain a healthy body weight or lose weight	Yes 1 *If C1=1 go to K1*<br>No 2 *If C1=1 go to K1*	H20f

Participant Identification Number └─┴─┴─┴─┴─┘

(for women only): Cervical Cancer Screening

The next question asks about cervical cancer prevention. Screening tests for cervical cancer prevention can be done in different ways, including Visual Inspection with Acetic Acid/vinegar (VIA), pap smear and Human Papillomavirus (HPV) test. VIA is an inspection of the surface of the uterine cervix after acetic acid (or vinegar) has been applied to it. For both pap smear and HPV test, a doctor or nurse uses a swab to wipe from inside your vagina, take a sample and send it to a laboratory. It is even possible that you were given the swab yourself and asked to swab the inside of your vagina. The laboratory checks for abnormal cell changes if a pap smear is done, and for the HP virus if an HPV test is done.

Question	Response	Code
Have you ever had a screening test for cervical cancer, using any of these methods described above?	Yes 1<br>No 2<br>Don't know 77	CX1

Khat use

Now I am going to ask you some questions about Khat chewing.

Question	Response		Code
Have you ever chewed Khat?<br>*(USE SHOWCARD)*	Yes 1<br>No 2 *If No, go to K14*		K1
Do you currently chew Khat?	Yes 1<br>No 2 If No, go to K8		K2
During the past 12 months, how frequently did you chew Khat?	Daily 1<br>5-6 days per week 2<br>3-4 days per week 3<br>1-2 days per week 4<br>1-3 days per month 5<br>Less than once a month 6		K3
How old were you when you **first started** chewing Khat?	Age (years) └─┴─┘<br>Don't know 77 *If Known, go to K5*		K4
Do you remember how long ago it was?<br>*(RECORD ONLY 1, NOT ALL 3)*<br>*Don't know 77*	In Years └─┴─┘ *If Known, go to K5*		K4a
	OR in Months └─┴─┘ *If Known, go to K5*		K4b
	OR in Weeks └─┴─┘		K4c
On average, **how many** bundles of Khat do you chew **each day/week?**<br>*(IF LESS THAN DAILY, RECORD WEEKLY)*<br>*(USE SHOWCARD)*<br>*Don't Know 77*	DAILY↓	WEEKLY↓	
	Bundles of Khat └─┴─┘	└─┴─┘	K5
During the past 12 months, have you tried to **stop chewing Khat**?	Yes 1<br>No 2		K6
During any visit to a doctor or other health worker in the past 12 months, were you advised to **quit chewing Khat**?	Yes 1 go to K9<br>No 2 *go to K9*<br>No visit during the past 12 months 3 *go to K9*		K7
How old were you when you **stopped** chewing Khat?	Age (years) └─┴─┘ *If Known, go to K10*		K8
How long ago did you stop chewing Khat?<br>(RECORD ONLY 1, NOT ALL 3)<br>Don't Know 77	Years ago └─┴─┘ **If Known, go to K10**		K8a
	OR Months ago └─┴─┘ **If Known, go to K10**		K8b
	OR Weeks ago └─┴─┘ **if known, go to K10**		K8c

Do you currently smoke tobacco products while chewing Khat?<br>(USE TOBACCO SHOWCARD)	Yes 1 If yes, go to K11<br>No 2	K9
In the **past**, did you **ever smoke** tobacco products while chewing Khat?	Yes 1 If K2=2 go to K13<br>No 2 If K2=2 go to K13	K10
Does one or more of your friends smoke tobacco products while you chew Khat together?	Yes 1<br>No 2	K11
Do you currently drink alcohol after you chew Khat?	Yes<br>No 1 If yes, go to K14	K12
In the **past**, did you **ever drink alcohol** while chewing Khat?	Yes 1<br>No 2	K13
During the past 12 months, have you had family problems or problems with your partner due to consumption of Khat by you or somebody else?	Yes 1<br>No 2	K14

Violence and Injury

CORE: Injury

The next questions ask about different experiences and behaviours that are related to road traffic injuries.

Question	Response	Code
In the past 12 months, have you been involved in a road traffic crash as a driver, passenger, pedestrian, or cyclist?	Yes (as driver) 1<br>Yes (as passenger) 2<br>Yes (as pedestrian) 3<br>Yes (as a cyclist) 4<br>No 5 *If No, go to V5*<br>Don't know 77 *If don't know, go to V5*<br>Refused 88 *If Refused, go to V5*	V3
Did you have any injuries in this road traffic crash which required medical attention?	Yes 1<br>No 2<br>Don't know 77<br>Refused 88	V4
The next questions ask about the most serious accidental injury you have had in the past 12 months.		
In the past 12 months, were you injured accidentally, other than road traffic crashes which required medical attention?	Yes 1<br>No 2 *If No, go to V11*<br>Don't know 77 *If don't know, go to V11*<br>Refused 88 *If Refused, go to V11*	V5
Please indicate which of the following the cause of this injury was.	Fall 1<br>Burn 2<br>Poisoning 3<br>Cut 4<br>Near-drowning 5<br>Animal bite 6<br>Other (specify) 7<br>Don't know 77<br>Refused 88	V6
	Other (please specify) ________________	V6other

**CORE: Violence**		
The following questions are about different experiences and behaviours that are related to violence.		
**Question**	**Response**	**Code**
In the past 12 months, how many times were you in a violent incident in which you were injured and required medical attention?	Never 1 *If never, go to V16*<br>Rarely (1- 2 times) 2<br>Sometimes (3 – 5 times) 3<br>Often (6 or more times) 4<br>Don't know 77 *If don't know, go to V16*<br>Refused 88 *If Refused, go to V16*	V11
The next questions ask about the most serious violent incidence you have had in the past 12 months.		
Please indicate which of the following caused your most serious injury in the last 12 months.<br>*(USE SHOWCARDS)*	Being shot with a firearm 1<br>A weapon (other than a firearm) was used by the person who injured me 2<br>Being injured without any weapon (slapped, pushed…) 3<br>Don't know 77<br>Refused 88	V12
Please indicate the relationship between yourself and the person(s) who caused your injury.	Intimate partner 1<br>Parent 2<br>Child, sibling, or other relative 3<br>Friend or acquaintance 4<br>Unrelated caregiver 5<br>Stranger 6<br>Official or legal authorities 7<br>Other (specify) 8<br>Refused 88	V13
	Other (please specify) ________________________	V13other
Since your 15th birthday, have you ever experienced a sex act involving vaginal, oral, or anal penetration **against your will**?	Never 1<br>Once 2<br>A few times (2 to 3 times) 3<br>Many times (4 or more times) 4<br>Don't know 77<br>Refused 88	V16

Participant Identification Number |__|__|__|__|__|

**P H Q – 9 mental health**)	Response	Code
**Over the <u>last 2 weeks</u>, how often have you been bothered by any of the following problems?**<br>*(Circle to indicate your answer*	Not at all (0)<br>Several days(1)<br>More than half the days (2)<br>Nearly every day(3)	
1.Little interest or pleasure in doing things	0 1 2 3	X7a
2.Feeling down, depressed, or hopeless	0 1 2 3	X7b
3.Trouble falling or staying asleep, or sleeping too much	0 1 2 3	X7c
4.Feeling tired or having little energy	0 1 2 3	X7d
5 Poor appetite or overeating	0 1 2 3	X7e
6 Feeling bad about yourself — or that you are a failure or have let yourself or your family down	0 1 2 3	X7f
7.Trouble concentrating on things, such as reading the newspaper or watching television	0 1 2 3	X7g
8 Moving or speaking so slowly that other people could have noticed? Or the opposite — being so fidgety or restless that you have been moving around a lot more than usual	0 1 2 3	X7h
9. Thoughts that you would be better off dead or of hurting yourself in some way	0 1 2 3	X7i
If you checked off <u>any</u> problems, how <u>difficult</u> have these problems made it for you to do your work, take care of things at home, or get along with other people?	Not difficult at all 1<br>Somewhat difficult 2<br>Very difficult 3<br>Extremely difficult 4	X8

Participant Identification Number └─┴─┴─┴─┴─┘

Step 2 Physical Measurements

**Blood Pressure**		
**Question**	**Response**	**Code**
Interviewer ID	└─┴─┘	M1
Device ID for blood pressure	└─┴─┘	M2
Cuff size used	Small 1<br>Medium 2<br>Large 3	M3
Reading 1	Systolic (mmHg) └─┴─┴─┘	M4a
	Diastolic (mmHg) └─┴─┴─┘	M4b
Reading 2	Systolic (mmHg) └─┴─┴─┘	M5a
	Diastolic (mmHg) └─┴─┴─┘	M5b
Reading 3	Systolic (mmHg) └─┴─┴─┘	M6a
	Diastolic (mmHg) └─┴─┴─┘	M6b
During the past two weeks, have you been treated for raised blood pressure with drugs (medication) prescribed by a doctor or other health worker?	Yes 1<br>No 2	M7
**Height and Weight**		
**For women:** Are you pregnant?	Yes 1 *If Yes, go to M 16 a-c*<br>No 2	M8
Interviewer ID	└─┴─┘	M9
Device IDs for height and weight	Height └─┴─┘<br>Weight └─┴─┘	M10a<br>M10b
Height	in Centimetres (cm) └─┴─┴─┘ . └─┘	M11
Weight<br>*If too large for scale 666.6*	in Kilograms (kg) └─┴─┴─┘ . └─┘	M12
**Waist**		
Device ID for waist	└─┴─┘	M13
Waist circumference	in Centimetres (cm) └─┴─┴─┘ . └─┘	M14

**Hip Circumference and Heart Rate**		
Hip circumference	in Centimeters (cm) └─┴─┴─┘ . └─┘	M15
Heart Rate		
Reading 1	Beats per minute └─┴─┴─┘	M16a
Reading 2	Beats per minute └─┴─┴─┘	M16b
Reading 3	Beats per minute └─┴─┴─┘	M16c

Participant Identification Number └─┴─┴─┴─┴─┘

Step 3 Biochemical Measurements

CORE: Blood Glucose		
**Question**	**Response**	**Code**
During the past 12 hours have you had anything to eat or drink, other than water?	Yes 1<br>No 2	B1
Technician ID	└─┴─┘	B2
Device ID	└─┴─┘	B3
Time of day blood specimen taken (24 hour clock)	Hours : minutes └─┴─┘ : └─┴─┘ hrs mins	B4
Fasting blood glucose	mg/dl └─┴─┴─┘ . └─┘	B5
Today, have you taken insulin or other drugs (medication) that have been prescribed by a doctor or other health worker for raised blood glucose?	Yes 1<br>No 2	B6
**CORE: Blood Lipids**		
Total cholesterol	mg/dl └─┴─┴─┘ . └─┘	B8
During the past two weeks, have you been treated for raised cholesterol with drugs (medication) prescribed by a doctor or other health worker?	Yes 1<br>No 2	B9
**CORE: Urinary sodium and creatinine**		
Had you been fasting prior to the urine collection?	Yes 1<br>No 2	B10
Technician ID	└─┴─┘	B11
Time of day urine sample taken (24 hour clock)	Hours : minutes └─┴─┘ : └─┴─┘ hrs mins	B13

EXPANDED: Triglycerides and HDL Cholesterol		
**Question**	**Response**	**Code**
HDL Cholesterol	mg/dl └─┴─┴─┘ . └─┘	B17
